# Endemic characteristics of SARS-CoV-2 infection

**DOI:** 10.1038/s41598-023-41841-8

**Published:** 2023-09-08

**Authors:** Igor Nesteruk

**Affiliations:** 1 Institute of Hydromechanics, National Academy of Sciences of Ukraine, Kyiv, Ukraine; 2 Igor Sikorsky Kyiv Polytechnic Institute, Kyiv, Ukraine

**Keywords:** Infectious diseases, Computational biology and bioinformatics, Environmental sciences, Diseases, Health care

## Abstract

The fourth year of the COVID-19 pandemic without decreasing trends in the global numbers of new daily cases, high numbers of circulating SARS-CoV-2 variants and re-infections together with pessimistic predictions for the Omicron wave duration force studies about the endemic stage of the disease. The global trends were illustrated with the use the accumulated numbers of laboratory-confirmed COVID-19 cases and deaths, the percentages of fully vaccinated people and boosters (additional vaccinations), and the results of calculation of the effective reproduction number provided by Johns Hopkins University. A new modified SIR model with re-infections was proposed and analyzed. The estimated parameters of equilibrium show that the global numbers of new daily cases will range between 300 thousand and one million, daily deaths—between one and 3.3 thousand.

## Introduction

The fourth year of the COVID-19 pandemic, the high numbers of circulating SARS-CoV-2 variants^1–3^ and re-infected persons^4–6^, the lack of decreasing trends in the global numbers of new daily cases^7,8^, and the expected very long duration of the Omicron wave^9^ make us think about the constant circulation of the pathogen, that is, about the endemic stage of the disease. To illustrate the global trends, we will use the accumulated numbers of laboratory-confirmed COVID-19 cases *V*_*j*_ and deaths *D*_*j*_, the percentages of fully vaccinated people *VC*_*j*_ and boosters *BC*_*j*,_ and the results of calculation of the effective reproduction number *R*_*j*_^10^. Information corresponding to day *t*_*j*_ (starting with January 22, 2020) is available in COVID-19 Data Repository by the Center for Systems Science and Engineering (CSSE) at Johns Hopkins University (JHU)^8^. We will use the version the JHU file updated on December 7, 2022 and the smoothed daily and annual characteristics^11^ (see formulae (8), (9) and Table 1):

**Table 1:** Averaged characteristics of the COVID-19 pandemic in 2020, 2021 and 2022.

Year	Average daily numbers of new cases, *DV*	Average daily numbers of deaths, *DD*	Average case fatality risk, *CFD*
2020	**2.4352e + 005** = (83,773,024–557)/344	**5.5281e + 003** = (1,901,671–17)/344	**0.0227** = (1,901,671–17)/(83,773,024–557)
2021	**5.6155e + 005** = (288,738,770–83,773,024)/365	**9.7745e + 003** = (5,469,351–1,901,671)/365	**0.0174** = (5,469,351–1,901,671)/(288,738,770–83,773,024)
2022	**1.0516e + 006** = (646,279,687–288,738,770)/340	**3.4567e + 003** = (6,644,643–5,469,351)/340	**0.0033** = (6,644,643–5,469,351)/(646,279,687–288,738,770)

Results of calculations are in [bold].

The classical SIR-model^12–17^ relates the numbers of susceptible *S*(*t*), infectious *I*(*t*) and removed persons *R*(*t*) over time *t* and can be successfully used for simulations of the first COVID-19 waves in individual countries and worldwide^11,18–20^. Nevertheless, this model yields only trivial equilibrium point $$I_{**} = R_{**} = 0$$ and cannot be used to estimate endemic characteristics. Numerous improvements of this model^9,11,21–24^ do not take into account re-infections. In this paper we will modify the classical SIR model by adding the re-infection terms, calculate a non-trivial equilibrium point, and estimate its stability and endemic characteristics of SARS-CoV-2 infection.

## Results

**Figure 1:**
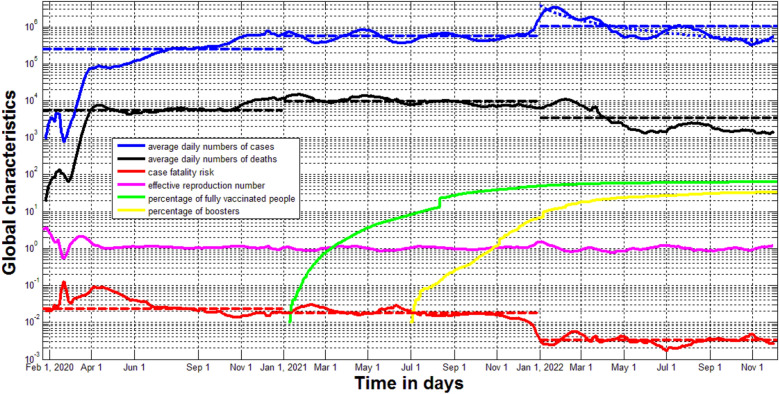
Global characteristics of the COVID-19 pandemic. Green, yellow and magenta lines respectively show *VC*_*j*_, *BC*_*j*_, *R*_*j*_ values listed in^8^. Solid blue, black and red represent calculations of *DV*_*i*_*, DD*_*i*_ and *CFR*_*i*_, respectively (Eqs. (8) and (9)). Corresponding dashed lines represent the average values for different years listed in Table 1. The dotted blue line shows the results of non-linear correlation (Eq. 7).

The solid lines in Fig. 1 illustrate the vaccination levels *VC*_*j*_ and *BC*_*j*_ (green and yellow colors, respectively), *R*_*j*_ values (magenta) and results of calculations of smoothed numbers of new daily cases *DV*_*i*_, deaths *DD*_*i*_, and the case fatality risk *CFR*_*i*_ = *DD*_*i*_*/DV*_*i*_ listed in Table 2 (blue, black and red, respectively). It can be seen that after October 2020, smoothed global numbers of new daily cases were never less than 300,000. After April 2020, the effective reproduction number^10^ was close to the critical value 1.0^25–28^. In 2022 we see the a sufficient drop in *CFI* figures.

**Table 2:** Accumulated numbers of COVID-19 cases and deaths form JHU dataset^8^, smoothed daily values of *DV*_*i*_ and *DD*_*i*_ calculated with the use of (8), (9) and case fatality risk CFR_i_ = *DD*_*i*_ /*DV*_*i*_.

Number of day, *t*_*j*_ (zero point is January 21, 2020)	Accumulated numbers of laboratory confirmed cases, *V*_*j*_	Accumu-lated numbers of COVID-19 related deaths, *D*_*j*_	Smoothed daily numbers of cases *DV*_*i*,_ calculated with the use of Eqs. (8) and (9)	Smoothed daily numbers of deaths *DD*_*i*,_ calculated with the use of Eqs. (8) and (9)	Case fatality risk, *CFR*_*i*_ = *DD*_*i*_*/DV*_*i*_
Year 2020					
1	557	17	–	–	–
2	657	18	–	–	–
3	944	26	–	–	–
4	1437	42	–	–	–
5	2120	56	942.285714285714	19.2857142857143	0.0204669496664645
6	2929	82	1183.07142857143	24.4285714285714	0.0206484332548451
7	5580	131	1398.85714285714	29.0000000000000	0.0207312091503268
8	6169	133	1804.85714285714	37.5714285714286	0.0208168434383410
9	8237	172	2258.92857142857	46.7142857142857	0.0206798418972332
10	9927	214	2519.78571428571	50.6428571428571	0.0200980809025711
11	12,038	260	2842.42857142857	56.8571428571429	0.0200030155299794
12	16,787	364	3146.00000000000	64.0714285714286	0.0203659976387249
13	19,887	428	3360.14285714286	69.3571428571429	0.0206411292036903
14	23,899	494	3540.42857142857	75.3571428571429	0.0212847516442723
15	27,644	566	3462	78	0.0225303292894281
16	30,806	636	3304.21428571429	80.7857142857143	0.0244492963531421
17	34,400	721	3128.50000000000	86.2857142857143	0.0275805383684559
18	37,131	808	2750.21428571429	83.9285714285714	0.0305171025634366
19	40,162	908	3369	92.2142857142857	0.0273714116100581
20	42,771	1015	4435	110.071428571429	0.0248188114028024
21	44,814	1115	4602.57142857143	118.857142857143	0.0258240734992861
22	45,232	1120	4500	123.142857142857	0.0273650793650794
23	60,384	1373	4398.42857142857	122.785714285714	0.0279158140894475
24	66,912	1525	4345.92857142857	125	0.0287625527998291
25	69,055	1668	4340.28571428571	135.714285714286	0.0312685142518597
26	71,238	1772	3303.92857142857	134.428571428571	0.0406874932439736
27	73,273	1870	1840.42857142857	114.714285714286	0.0623302025925638
28	75,155	2010	1391.92857142857	108.785714285714	0.0781546672140405
29	75,655	2125	1236.07142857143	106.785714285714	0.0863912164114418
30	76,216	2250	1002.64285714286	104.571428571428	0.104295789698653
31	76,846	2254	824.428571428573	104.714285714286	0.127014382256108
32	78,608	2462	784.714285714283	96.5714285714287	0.123065720007283
33	78,990	2473	874.357142857138	86.8571428571429	0.0993382893554455
34	79,558	2633	987	84.9285714285713	0.0860471848313793
35	80,412	2713	1051.50000000000	79	0.0751307655729910
36	81,384	2774	1201.92857142857	72.2142857142858	0.0600820110536638
37	82,728	2817	1445.42857142857	70.2857142857142	0.0486262107135797
38	84,152	2876	1671.85714285715	64.8571428571429	0.0387934717593779
39	86,023	2946	1893.85714285715	66.9285714285713	0.0353398204722033
40	88,402	3000	2087.92857142857	73.1428571428571	0.0350313023844548
41	90,382	3090	2364.57142857143	80.7857142857145	0.0341650555824070
42	92,994	3164	2705	87.1428571428571	0.0322154739899657
43	95,316	3260	2971.78571428572	103	0.0346592957577214
44	98,027	3355	3244.50000000000	123.857142857143	0.0381744930982102
45	101,957	3469	3567.14285714286	145.642857142857	0.0408289947937525
46	106,088	3573	4090.57142857143	178.285714285714	0.0435845498358595
47	109,942	3815	4747.64285714286	212.714285714286	0.0448041885446913
48	114,265	4009	5698.28571428571	256.642857142857	0.0450386081026876
49	119,051	4284	6842.64285714286	307.428571428572	0.0449283380481643
50	126,527	4636	7852.35714285715	360.714285714286	0.0459370707612819
51	133,283	4957	9111.14285714285	428.214285714286	0.0469989651604002
52	146,477	5460	10,584.8571428571	506.571428571428	0.0478581261640619
53	157,365	5886	12,047.1428571429	590.714285714286	0.0490335586386813
54	168,598	6552	14,219.6428571429	692.357142857143	0.0486901921386412
55	183,165	7267	16,838.7142857143	827.571428571428	0.0491469487829916
56	198,339	8118	19,544.0714285714	1002.85714285714	0.0513126011907155
57	215,899	9072	22,721.5000000000	1184.71428571429	0.0521406723021933
58	242,986	10,214	26,359.2857142857	1366.50000000000	0.0518413137143322
59	272,516	11,789	30,265.2142857143	1577.64285714286	0.0521272653895444
60	304,943	13,597	34,595.1428571429	1834.21428571429	0.0530194164333556
61	339,121	15,427	39,426.7857142857	2129.07142857143	0.0540006340866887
62	381,672	17,523	44,291.5000000000	2427.21428571429	0.0548009050430508
63	423,545	19,949	49,400.0714285714	2734.64285714286	0.0553570628151203
64	475,025	22,920	53,647.3571428572	3036.78571428571	0.0566064364773661
65	535,835	26,173	56,845.7142857143	3341.14285714286	0.0587756332931242
66	599,748	29,811	61,061.2857142857	3699.28571428571	0.0605831611799855
67	669,312	33,860	66,161.9285714286	4157.50000000000	0.0628382529011613
68	725,815	37,679	70,193.7857142857	4633.35714285715	0.0660080817084948
69	790,818	42,047	73,185.5000000000	5051.28571428571	0.0690203074965084
70	869,257	47,215	73,956.6428571428	5420.71428571428	0.0732958403234327
71	955,580	53,859	74,364.3571428572	5735.78571428571	0.0771308451341416
72	1,037,993	60,101	76,140.0714285714	6032.9285714285	0.0792346061441273
73	1,122,187	66,601	76,151.500000000	6419.14285714286	0.0842943718395942
74	1,182,266	72,960	75,174.5714285715	6699.35714285714	0.0891173307083321
75	1,253,962	78,880	75,215.8571428572	6876.92857142857	0.0914292388952937
76	1,328,632	85,307	75,714.0714285714	7102	0.0938002654724495
77	1,397,564	93,823	76,973.5000000000	7235.07142857143	0.0939943152977509
78	1,479,717	101,042	81,421.2857142858	7322	0.0899273443764266
79	1,566,878	109,195	84,565.4285714285	7370.50000000000	0.0871573658942020
80	1,653,299	116,935	85,481.5000000000	7293.71428571429	0.0853250619808297
81	1,728,783	123,917	86,322.7142857143	7321.28571428571	0.0848129692731096
82	1,847,343	130,431	86,565.5000000000	7399.92857142857	0.0854835768456090
83	1,919,167	136,943	87,189.4285714285	7450.07142857143	0.0854469578553102
84	2,003,770	144,299	87,487.7857142857	7492.42857142858	0.0856397097064163
85	2,082,029	153,064	84,665.6428571430	7423.21428571429	0.0876768194890995
86	2,176,483	160,772	81,980.7857142858	7393.50000000000	0.0901857665254314
87	2,264,346	169,659	81,704.4285714284	7382.07142857144	0.0903509339413323
88	2,342,565	176,087	81,363.6428571427	7264.28571428571	0.0892817167372933
89	2,418,880	182,186	80,790.9285714286	7126.71428571428	0.0882118130306353
90	2,495,361	188,697	79,643.2142857143	6948.92857142857	0.0872507298173550
91	2,571,438	195,894	79,688.5714285714	6775.14285714286	0.0850202574307125
92	2,653,452	203,169	79,644	6656.50000000000	0.0835781728692683
93	2,736,133	210,441	78,857.6428571430	6488.85714285715	0.0822857101449537
94	2,819,701	217,275	78,444.8571428570	6372	0.0812290344081558
95	2,902,850	223,323	78,086.1428571430	6310.21428571429	0.0808109359077795
96	2,973,611	228,141	77,823.2857142859	6216.42857142858	0.0798787729709983
97	3,044,637	233,586	78,281.4285714284	6054.71428571429	0.0773454751172512
98	3,120,390	240,213	78,315	5963.21428571429	0.0761439607446120
99	3,197,706	247,193	78,194.9285714286	5922.07142857142	0.0757347252150987
100	3,281,405	253,447	78,896.6428571427	5840.42857142857	0.0740263255814796
101	3,370,369	259,035	79,645.2857142857	5763.14285714287	0.0723601253414696
102	3,448,592	265,048	80,861.2857142857	5713.35714285713	0.0706562737951588
103	3,522,598	269,325	82,145.2857142857	5670.35714285713	0.0690283939431355
104	3,600,203	274,168	82,595.5000000000	5668.92857142858	0.0686348356923631
105	3,679,858	280,315	83,128.8571428573	5616.71428571429	0.0675663599712666
106	3,770,296	287,078	83,644.9285714286	5542.78571428571	0.0662656518327043
107	3,858,849	292,947	83,629.1428571427	5492.57142857142	0.0656777200019132
108	3,949,262	298,900	83,936.2857142857	5408.35714285716	0.0644340775486170
109	4,033,503	303,817	83,915.2142857143	5284	0.0629683192133556
110	4,108,716	308,155	83,991.3571428573	5180.85714285713	0.0616832174058723
111	4,184,893	312,234	84,920.3571428570	5158.35714285716	0.0607434697216302
112	4,270,276	317,966	86,033.4285714284	5117.42857142858	0.0594818625318400
113	4,354,691	323,403	86,947.7142857146	5057.71428571429	0.0581696060358112
114	4,450,333	329,154	88,097.4285714286	5047.42857142858	0.0572937105347651
115	4,546,663	334,910	89,753.3571428568	5015.50000000000	0.0558809181033421
116	4,640,570	339,451	91,983.8571428573	4958.78571428571	0.0539093039617199
117	4,718,917	343,329	94,220.8571428573	4905.42857142855	0.0520630858196393
118	4,808,056	347,724	95,737.2857142855	4850.64285714284	0.0506661832007532
119	4,903,660	352,693	97,245.7142857146	4840.71428571429	0.0497781760488893
120	5,009,081	358,099	99,035.7142857146	4800.50000000000	0.0484724125495852
121	5,115,035	363,134	100,030.857142857	4573.64285714287	0.0457223199698382
122	5,222,283	368,839	99,732.7142857141	4367.14285714287	0.0437884688932850
123	5,326,390	373,292	99,295.8571428573	4340.28571428571	0.0437106425099018
124	5,419,597	376,695	100,093.642857143	4323.92857142858	0.0431988330927255
125	5,507,808	378,389	102,112.642857143	4281.64285714284	0.0419305850611753
126	5,600,166	383,168	105,338.357142857	4235.85714285713	0.0402119157517576
127	5,702,717	388,388	108,237.285714286	4221.14285714287	0.0389989718356891
128	5,822,710	393,380	109,788.785714286	4359.21428571429	0.0397054604197800
129	5,944,185	398,536	112,821.357142857	4540.07142857142	0.0402412410517511
130	6,079,225	402,897	115,613.428571429	4603.28571428571	0.0398161854653562
131	6,182,084	406,186	117,544.500000000	4670.07142857145	0.0397302419813045
132	6,282,364	409,927	119,717.357142857	4701.21428571429	0.0392692788908162
133	6,405,109	415,191	120,172.928571428	4678.35714285713	0.0389302083129015
134	6,516,362	420,811	120,347.428571429	4660.28571428571	0.0387236002431056
135	6,654,688	426,338	121,127.642857143	4709	0.0388763447296151
136	6,788,250	431,395	121,557.714285715	4744.35714285713	0.0390296672715134
137	6,917,581	435,535	123,642.500000000	4715	0.0381341367248317
138	7,028,592	438,792	125,342.714285714	4673.64285714287	0.0372869127956610
139	7,131,643	443,247	124,755.357142857	4623.64285714287	0.0370616778552312
140	7,257,638	448,292	124,646.857142857	4619.28571428571	0.0370589826343681
141	7,394,828	453,720	126,468.571428572	4674.71428571426	0.0369634465931680
142	7,531,020	458,860	129,526.071428571	4684.42857142858	0.0361659125438068
143	7,658,493	463,604	132,367.928571428	4804.85714285716	0.0362992546209135
144	7,792,394	467,996	134,077	4956.50000000000	0.0369675634150525
145	7,924,339	471,777	134,999.571428572	4975.35714285713	0.0368546143532729
146	8,049,261	475,844	139,314.928571429	5106.21428571429	0.0366523124124222
147	8,193,171	482,963	146,849.857142857	5228.14285714287	0.0356019607976662
148	8,336,373	488,440	147,785.142857143	5296.14285714284	0.0358367746226181
149	8,479,469	493,795	146,251.500000000	5343.42857142858	0.0365358890091970
150	8,660,453	500,156	149,228.928571428	5218.35714285716	0.0349688039230236
151	8,846,332	504,638	152,981.428571429	5109.07142857142	0.0333966774678531
152	8,939,393	509,281	157,601.285714286	5093.07142857142	0.0323161794365345
153	9,081,728	513,148	161,022.928571428	4984.28571428571	0.0309538881108769
154	9,249,909	518,716	161,166.285714285	4911.14285714287	0.0304725199527730
155	9,421,375	524,214	165,874.142857143	4856.07142857142	0.0292756384143227
156	9,600,885	529,324	172,000.642857143	4797.64285714287	0.0278931681733749
157	9,793,358	534,407	173,854.357142857	4789.71428571432	0.0275501538438785
158	9,969,755	539,143	178,085.642857143	4755.78571428568	0.0267050484137045
159	10,138,208	542,761	183,022.500000000	4772.92857142852	0.0260783705360189
160	10,290,922	546,835	185,565.571428572	4812.71428571432	0.0259353836418241
161	10,474,676	552,085	187,241.285714285	4828.21428571432	0.0257860560361766
162	10,689,807	557,426	189,460.571428571	4847.71428571426	0.0255869295081373
163	10,894,768	562,933	191,841.142857143	4877.78571428568	0.0254261710581968
164	11,097,393	568,176	194,797.785714286	4971.71428571426	0.0255224373700344
165	11,287,098	572,969	196,651.928571429	5071.50000000000	0.0257892207660598
166	11,473,313	576,803	198,062	5116.21428571432	0.0258313774763171
167	11,641,593	581,082	201,639.571428571	5176.35714285716	0.0256713357709691
168	11,851,174	587,442	205,466	5236.28571428568	0.0254849255559834
169	12,066,436	593,070	207,595.142857144	5294.57142857142	0.0255043126525117
170	12,291,007	598,916	209,946	5339.42857142858	0.0254323900975898
171	12,524,108	604,662	212,633.071428571	5322.57142857142	0.0250317196323781
172	12,736,907	609,791	214,485.571428571	5316.42857142858	0.0247868821012936
173	12,929,836	614,105	216,974.142857143	5361	0.0247080132655702
174	13,124,314	618,532	218,735.785714286	5475.28571428574	0.0250315040879391
175	13,345,316	624,508	220,654.285714286	5666.42857142858	0.0256801201621152
176	13,575,092	630,434	223,617.142857143	5809.35714285710	0.0259790330411671
177	13,819,989	636,606	225,949.500000000	5817.64285714290	0.0257475358747990
178	14,057,427	643,626	228,499.785714285	5810.57142857148	0.0254292204712917
179	14,292,748	650,157	233,327.357142856	5943.28571428568	0.0254718768817446
180	14,504,635	655,070	239,064.571428572	6083.21428571426	0.0254459046330577
181	14,712,808	659,014	244,836.500000000	6076.57142857142	0.0248188951752350
182	14,955,819	665,374	249,142.857142856	6003.35714285716	0.0240960435779818
183	15,231,172	672,774	250,152.714285715	5933.71428571432	0.0237203673870077
184	15,510,813	679,431	252,019.071428572	5993.07142857142	0.0237802297841971
185	15,794,314	685,873	255,382.071428572	6131.21428571426	0.0240080059317285
186	16,043,861	691,957	256,507.071428572	6118.64285714284	0.0238537004967002
187	16,255,660	696,342	256,293.285714285	6073.14285714284	0.0236960669500845
188	16,490,050	701,645	256,727.214285714	6091.64285714284	0.0237280760206567
189	16,753,926	708,580	256,773.928571429	6096.85714285716	0.0237440661393360
190	17,024,164	715,229	257,881.428571429	6112.50000000000	0.0237027537572638
191	17,305,927	722,000	257,201.928571427	6099.50000000000	0.0237148299543412
192	17,593,381	728,587	255,282.071428571	6082.78571428574	0.0238277043125127
193	17,839,629	734,599	255,735.857142858	6153.78571428574	0.0240630538988052
194	18,070,232	739,275	256,440.785714285	6212.21428571426	0.0242247514115622
195	18,276,305	744,105	256,436	6228.35714285710	0.0242881543264483
196	18,541,620	751,279	257,433.071428573	6242	0.0242470789217612
197	18,816,772	758,683	258,959.571428571	6281	0.0242547512932245
198	19,103,490	765,517	261,069.357142856	6357.14285714290	0.0243503984026141
199	19,385,922	772,267	263,025.928571429	6356.35714285716	0.0241662758397258
200	19,651,151	778,307	263,038	6284	0.0238900843224173
201	19,884,144	783,501	263,274.142857142	6248.64285714284	0.0237343583738624
202	20,117,364	788,879	265,276.571428571	6233.92857142858	0.0234997328933254
203	20,382,924	795,494	265,879.285714287	6221.57142857148	0.0233999854928795
204	20,658,000	802,444	263,081.285714285	6193.14285714284	0.0235407959191320
205	20,948,100	809,237	259,988.928571427	6112.71428571426	0.0235114407344269
206	21,255,184	815,822	258,097.714285715	6091.64285714284	0.0236020798324443
207	21,504,199	821,854	257,829.785714285	6084.28571428574	0.0235980714851466
208	21,714,234	826,658	256,669.857142858	6020.71428571432	0.0234570368049229
209	21,927,119	831,300	252,115.928571429	5936.50000000000	0.0235467073962290
210	22,186,537	838,356	249,929.214285715	5859.14285714284	0.0234432092058065
211	22,464,004	844,762	250,445.214285715	5807.71428571426	0.0231895598495592
212	22,735,474	851,209	251,011.500000000	5788.07142857142	0.0230589890446112
213	22,997,433	856,961	251,082.714285715	5767.07142857142	0.0229688110747795
214	23,260,959	862,743	250,368.142857142	5742.57142857142	0.0229365100648931
215	23,463,707	867,077	251,796.500000000	5736.42857142858	0.0227820028134965
216	23,691,807	871,914	254,493.214285715	5724.71428571432	0.0224945655300942
217	23,937,007	878,481	255,861.428571429	5729.92857142858	0.0223946555892419
218	24,218,688	885,033	256,873.071428571	5726	0.0222911649249783
219	24,505,941	891,248	260,672.285714285	5692.64285714284	0.0218383125829585
220	24,789,871	897,068	264,807.357142858	5666.42857142852	0.0213983049133019
221	25,050,581	902,855	266,369.928571427	5651.21428571432	0.0212156616778119
222	25,270,308	907,129	266,252.428571427	5624.85714285716	0.0211260313118541
223	25,534,618	911,559	267,391.857142858	5618.07142857142	0.0210106301986970
224	25,801,499	918,166	269,590	5570.14285714284	0.0206615336516297
225	26,083,375	924,465	271,178.785714285	5510.78571428568	0.0203215959529071
226	26,368,788	930,564	268,481.214285715	5871.78571428574	0.0218703782680194
227	26,670,510	936,405	263,666.500000000	6136.71428571432	0.0232745315984940
228	26,944,202	941,500	262,454.928571429	6028.28571428574	0.0229688417249330
229	27,173,190	945,635	264,055	6019.07142857142	0.0227947640778301
230	27,390,473	955,258	266,456.285714285	6023.57142857142	0.0226062275559540
231	27,636,975	960,381	268,572.714285715	6032.14285714284	0.0224599988617075
232	27,922,268	966,646	271,060.642857144	6017.28571428568	0.0221990387496312
233	28,226,665	972,650	276,039.500000000	5646.64285714284	0.0204559233629348
234	28,543,021	978,649	282,032.142857142	5391.07142857142	0.0191150957970850
235	28,831,709	983,706	285,867.071428571	5465.71428571432	0.0191197756999446
236	29,080,532	987,671	288,069.357142858	5412.21428571432	0.0187878861514254
237	29,347,684	992,275	289,600.785714285	5376.71428571426	0.0185659519964798
238	29,628,214	998,839	290,648.071428571	5381.85714285716	0.0185167481635253
239	29,933,168	1,004,708	291,410.571428573	5398.92857142858	0.0185268796013871
240	30,248,736	1,010,359	291,343.357142856	5388.07142857142	0.0184938880412827
241	30,575,361	1,016,214	291,096.571428571	5346.07142857142	0.0183652847655859
242	30,868,442	1,021,487	291,843.214285715	5365.21428571426	0.0183838925254631
243	31,123,547	1,025,475	292,701.500000000	5414.50000000000	0.0184983677910773
244	31,383,476	1,029,904	293,323.714285715	5426.85714285716	0.0185012560476821
245	31,667,774	1,036,055	293,035.428571429	5449.85714285716	0.0185979462259074
246	31,979,413	1,042,605	291,924.785714285	5453.64285714284	0.0186816711838935
247	32,300,312	1,048,265	291,545.642857142	5424.07142857148	0.0186045360699467
248	32,630,317	1,054,284	291,638.571428573	5395.64285714290	0.0185011290883531
249	32,915,982	1,059,715	292,450.285714285	5380.42857142852	0.0183977545389886
250	33,162,954	1,063,598	293,242.428571427	5602.50000000000	0.0191053526165821
251	33,425,708	1,067,718	293,076.071428571	5809.21428571432	0.0198215236658451
252	33,708,482	1,073,780	293,868.428571429	5738.57142857136	0.0195276895053615
253	34,033,009	1,080,206	295,664.428571429	5702.28571428568	0.0192863434463104
254	34,352,110	1,089,099	300,102.714285713	5924.71428571432	0.0197422882355996
255	34,681,584	1,094,779	305,952.357142858	6122.57142857148	0.0200115190670444
256	34,978,873	1,099,560	310,094.642857146	6072.35714285716	0.0195822703898002
257	35,239,365	1,103,585	314,730.571428571	5857.21428571420	0.0186102489476257
258	35,550,735	1,110,677	319,809.142857142	5714.42857142852	0.0178682464183994
259	35,866,788	1,116,537	326,170.857142858	5759.78571428580	0.0176587993321644
260	36,216,028	1,122,462	332,391.428571429	5780.71428571432	0.0173912856614835
261	36,575,319	1,128,844	332,934.142857142	5581.35714285704	0.0167641476928725
262	36,935,703	1,135,036	331,646.642857142	5332.35714285716	0.0160784294299463
263	37,291,146	1,139,940	334,129.071428571	5312.07142857148	0.0158982617281989
264	37,580,572	1,144,135	339,777.857142858	5317	0.0156484593925863
265	37,870,606	1,148,266	346,759.071428571	5306.71428571432	0.0153037504220202
266	38,189,970	1,153,601	351,522.500000000	5367.50000000000	0.0152692928617656
267	38,570,653	1,159,767	355,690.21428571	5411	0.0152126760385190
268	38,977,584	1,165,977	364,738.857142858	5446.57142857136	0.0149327973203527
269	39,388,065	1,172,197	375,763.642857142	5589.78571428568	0.0148758024373604
270	39,760,099	1,177,924	384,922.571428571	5735.21428571432	0.0148996569996639
271	40,091,282	1,181,905	394,486.142857142	5770.28571428568	0.0146273470406166
272	40,466,240	1,186,748	405,802.500000000	5815.64285714284	0.0143312149559030
273	40,855,027	1,193,376	417,768.571428571	5892.64285714284	0.0141050410685342
274	41,294,512	1,200,285	425,752.857142855	5933.71428571432	0.0139369922859340
275	41,776,531	1,206,243	435,449.214285716	6032.14285714296	0.0138526897264879
276	42,270,353	1,213,350	448,861.214285716	6159.07142857148	0.0137215496294830
277	42,726,571	1,219,268	460,417.571428571	6234.14285714284	0.0135401931724711
278	43,085,350	1,223,633	471,241.357142858	6344.71428571420	0.0134638316216180
279	43,568,461	1,229,470	481,759.642857142	6483.78571428568	0.0134585489059081
280	44,036,863	1,236,881	488,452.785714284	6583.50000000000	0.0134782730133736
281	44,558,522	1,244,058	495,319.357142858	6706.35714285716	0.0135394610490115
282	45,109,900	1,251,296	505,722	6812.07142857148	0.0134699922656548
283	45,681,619	1,259,070	517,316.571428571	7001.42857142852	0.0135341277626078
284	46,153,644	1,265,717	522,138	7352.50000000000	0.0140815263397799
285	46,592,748	1,271,073	524,879.571428571	7654.78571428580	0.0145838895833794
286	47,141,171	1,277,399	533,394.714285716	7861.71428571432	0.0147390179826625
287	47,706,585	1,286,972	546,131.642857146	8044.35714285704	0.0147297034480041
288	48,198,732	1,296,902	558,897.142857142	8200.28571428568	0.0146722627214821
289	48,818,004	1,305,619	559,837.214285713	8364.50000000000	0.0149409503094076
290	49,441,041	1,314,811	557,474.714285713	8466.85714285716	0.0151878765545549
291	50,040,065	1,322,597	567,329.214285713	8514.28571428580	0.0150076630991154
292	50,530,887	1,328,997	578,281.285714287	8620.50000000000	0.0149071052668634
293	51,040,753	1,336,578	582,479.285714287	8744.85714285716	0.0150131641713807
294	51,611,649	1,346,329	584,306.214285713	8894.28571428568	0.0152219598163243
295	52,236,277	1,356,745	583,376.571428571	9030.78571428568	0.0154801995084772
296	52,876,397	1,366,463	584,470.285714287	9104.71428571432	0.0155777197032136
297	53,537,358	1,376,395	587,838.857142858	9226.07142857136	0.0156948988935742
298	54,124,035	1,385,533	589,893	9388.85714285716	0.0159162036892405
299	54,614,189	1,392,492	590,701.071428571	9566	0.0161943163178378
300	55,140,035	1,400,549	592,624.142857142	9797.71428571420	0.0165327626351464
301	55,742,111	1,411,523	593,969.714285713	9939.57142857148	0.0167341384409211
302	56,364,317	1,422,995	594,934.571428571	10,002.1428571428	0.0168121728631864
303	57,018,172	1,434,137	596,101.142857146	10,100.8571428572	0.0169448712922159
304	57,692,321	1,445,889	595,888.571428571	10,259.4285714286	0.0172170252348235
305	58,284,648	1,455,193	594,705.928571429	10,409.4285714285	0.0175034888191438
306	58,782,660	1,462,862	589,710.428571429	10,434.0714285714	0.0176935508056859
307	59,316,980	1,471,591	586,593.928571425	10,402.7857142858	0.0177342198880586
308	59,907,606	1,484,113	589,180.500000000	10,401.3571428572	0.0176539399095136
309	60,524,705	1,496,137	590,075.142857146	10,415.5714285714	0.0176512628173746
310	61,113,730	1,507,072	587,926.500000000	10,437.5000000000	0.0177530694738203
311	61,809,078	1,518,593	587,677.857142855	10,455.1428571428	0.0177906019940468
312	62,416,418	1,528,108	591,054.928571429	10,476.1428571428	0.0177244827015714
313	62,911,942	1,535,765	599,962.071428575	10,629	0.0177161199118658
314	63,418,669	1,544,813	607,289.857142858	10,823.5000000000	0.0178226260041980
315	64,033,407	1,557,263	609,847	10,968.8571428572	0.0179862443249818
316	64,673,673	1,569,653	615,779.214285713	11,077.5714285715	0.0179895182746971
317	65,364,231	1,582,362	621,257.50000000	11,094.1428571428	0.0178575596385441
318	66,060,635	1,594,832	624,404.571428571	11,050	0.0176968595452765
319	66,702,719	1,605,433	627,316.142857138	11,032.3571428572	0.0175865985093414
320	67,246,550	1,613,526	686,588.785714287	11,023.6428571428	0.0160556698368944
321	67,781,666	1,622,370	744,735.571428575	11,054.0714285715	0.0148429480914511
322	68,412,074	1,634,406	745,858.500000000	11,121.4285714286	0.0149109094706686
323	69,077,432	1,646,963	745,948.500000000	11,149.4285714285	0.0149466465465492
324	70,572,715	1,659,383	746,731.357142858	11,186.0714285714	0.0149800478064447
325	71,278,449	1,672,568	749,173.071428575	11,296	0.0150779578588697
326	71,926,924	1,683,397	754,195	11,478.6428571428	0.0152197281301823
327	72,465,624	1,691,654	703,870.928571425	11,629.9285714286	0.0165228141969618
328	73,016,831	1,700,847	650,937.142857142	11,673.1428571428	0.0179328265182506
329	73,665,332	1,714,073	651,111.785714284	11,693.2142857142	0.0179588429241632
330	74,382,904	1,727,997	650,771.785714284	11,732.0714285715	0.0180279349629370
331	75,121,436	1,741,168	651,031.857142858	11,761.3571428572	0.0180657167753257
332	75,842,848	1,754,207	650,886.357142858	11,828.7142857143	0.0181732404680255
333	76,478,090	1,765,463	648,551.428571433	11,862.5000000000	0.0182907622702020
334	77,025,263	1,773,837	642,783.928571433	11,780.8571428572	0.0183278651179717
335	77,571,638	1,783,323	625,070.285714284	11,425.5000000000	0.0182787444246271
336	78,222,934	1,797,199	601,433.357142851	10,902.3571428570	0.0181272904360500
337	78,905,022	1,810,946	582,702.857142858	10,647.2857142857	0.0182722387298598
338	79,598,293	1,823,151	569,767.428571433	10,633.2142857143	0.0186623765285685
339	80,116,975	1,832,181	567,861.071428567	10,715.7857142858	0.0188704354875535
340	80,624,030	1,840,122	572,402.928571425	10,913.5000000000	0.0190661148908469
341	81,037,163	1,848,240	585,940.071428575	11,125	0.0189865833426894
342	81,536,482	1,857,785	601,244.142857142	11,297.7142857143	0.0187905602406820
343	82,208,145	1,872,758	611,397.857142858	11,442.2142857142	0.0187148419838845
344	82,933,452	1,888,176	624,349.571428575	11,495.2142857143	0.0184115034457573
345	83,773,024	1,901,671	636,166.571428567	11,513.2142857143	0.0180977982855346
Year 2021					
346	84,359,662	1,911,829	645,269.642857142	11,573.7142857142	0.0179362448145984
347	84,940,913	1,920,665	654,903.285714291	11,569.8571428572	0.0176665125908448
348	85,461,174	1,928,630	661,763.785714284	11,639.4285714286	0.0175884942976525
349	86,018,803	1,938,580	681,705.857142851	12,105.5000000000	0.0177576587807772
350	86,759,599	1,953,995	711,971.785714291	12,789.4285714285	0.0179633924097111
351	87,550,644	1,968,917	730,263.428571433	13,225.8571428572	0.0181110769420975
352	88,420,525	1,983,882	740,135.642857142	13,363.2857142857	0.0180551846722304
353	89,256,043	1,999,095	740,745.714285716	13,499.3571428572	0.0182240097816486
354	90,012,137	2,012,451	733,876	13,723.0714285715	0.0186994416339702
355	90,613,638	2,022,006	722,550.714285709	13,888.1428571428	0.0192209938798168
356	91,228,238	2,032,290	710,947.642857142	13,977.8571428572	0.0196608811960938
357	91,920,604	2,049,276	700,166.214285716	14,062.4285714285	0.0200844146497050
358	92,663,903	2,065,759	687,792.142857142	14,130.8571428572	0.0205452436315374
359	93,422,976	2,081,474	675,556.071428575	14,162.4285714286	0.0209641052318275
360	94,206,859	2,097,193	661,485.714285716	14,082.7857142857	0.0212896294056668
361	94,863,648	2,111,227	650,364	14,080.8571428570	0.0216507327325268
362	95,391,217	2,121,062	639,908.214285716	14,285.7142857143	0.0223246302622642
363	95,908,444	2,131,508	624,865	14,426.3571428573	0.0230871582547547
364	96,501,198	2,147,217	610,544.285714284	14,489.2857142857	0.0237317522304455
365	97,188,405	2,164,950	600,130.357142851	14,521.6428571427	0.0241974809044464
366	97,857,189	2,182,283	592,898.214285716	14,565.8571428570	0.0245672137171218
367	98,520,756	2,198,353	588,103	14,735	0.0250551348998390
368	99,097,371	2,212,917	579,694.357142851	14,796.3571428573	0.0255244112014206
369	99,559,319	2,222,675	569,700.214285716	14,683.5714285716	0.0257742073117906
370	100,040,917	2,233,817	560,535.857142858	14,604.6428571427	0.0260547878802704
371	100,602,167	2,251,198	550,790.714285716	14,491.7857142857	0.0263108751444350
372	101,203,157	2,268,118	541,702.500000000	14,388.0714285716	0.0265608363051151
373	101,818,240	2,284,685	534,276.071428567	14,309.2857142857	0.0267825689367390
374	102,407,207	2,300,416	524,681.142857142	14,064.9285714284	0.0268066210552910
375	102,921,990	2,313,739	512,432.428571433	13,809.0714285714	0.0269480826322185
376	103,318,535	2,323,286	496,784.785714291	13,473.9285714286	0.0271222649301859
377	103,761,566	2,333,536	482,797.571428567	13,317.8571428573	0.0275847641558150
378	104,227,054	2,348,388	473,360.785714284	13,317	0.0281328753920863
379	104,752,324	2,364,255	464,456.071428575	13,085.1428571427	0.0281730472741920
380	105,224,060	2,377,183	453,876.857142858	12,908.7857142857	0.0284411630845118
381	105,760,553	2,394,368	442,351.142857142	12,851.3571428573	0.0290523882448918
382	106,195,695	2,406,225	432,130.428571425	12,699.7142857143	0.0293886138212904
383	106,547,215	2,413,992	424,074.642857142	12,623.8571428570	0.0297680074852050
384	106,887,162	2,423,553	414,441	12,384.2142857143	0.0298817305375538
385	107,294,374	2,438,290	402,898.714285716	11,993.2142857143	0.0297673183370084
386	107,734,830	2,452,149	395,929	11,862.1428571430	0.0299602778708884
387	108,178,599	2,466,023	388,646.071428575	11,690.3571428570	0.0300796997635559
388	108,608,188	2,478,907	381,037.928571433	11,306	0.0296715868742722
389	108,988,642	2,489,591	373,785.357142851	10,867.1428571430	0.0290732171538486
390	109,297,274	2,496,696	367,566.071428567	10,553.5000000000	0.0287118448092426
391	109,578,148	2,504,514	363,786.928571433	10,330.9285714286	0.0283982951558966
392	109,937,919	2,515,613	361,985.071428575	10,130.5714285714	0.0279861580716328
393	110,324,280	2,526,966	362,387.071428567	9995.64285714272	0.0275827799753972
394	110,735,074	2,538,955	364,217.928571425	9932.71428571432	0.0272713491196699
395	111,144,730	2,550,608	367,622.857142858	9919.57142857136	0.0269830105387506
396	111,519,891	2,559,718	374,162.928571433	10,024.9285714284	0.0267929498245696
397	111,839,444	2,566,508	381,362.142857142	10,025.7142857143	0.0262892226548820
398	112,135,029	2,573,760	386,682.785714284	9870.14285714296	0.0255251674545345
399	112,527,758	2,585,241	390,529.571428575	9764.78571428568	0.0250039598245164
400	112,972,722	2,597,687	391,228	9716.85714285704	0.0248368141923815
401	113,425,702	2,608,594	391,097.500000000	9721.35714285728	0.0248566077330008
402	113,867,661	2,619,151	386,131.214285716	9658.71428571432	0.0250140727513611
403	114,264,374	2,627,882	380,232.642857142	9503.50000000000	0.0249939088043279
404	114,572,153	2,634,380	380,401.285714284	9404.42857142864	0.0247223890260251
405	114,877,685	2,641,987	381,302.928571433	9367.28571428568	0.0245665191961169
406	115,190,939	2,652,236	383,049.714285716	9332.71428571432	0.0243642376894010
407	115,632,798	2,663,741	389,046.714285709	9273.57142857136	0.0238366527412978
408	116,091,244	2,674,202	393,807.285714284	9213.92857142864	0.0233970495358320
409	116,540,360	2,684,685	401,005.285714291	9201.71428571432	0.0229466159512680
410	116,954,371	2,693,006	409,418.785714284	9122.92857142841	0.0222826330636302
411	117,328,810	2,699,086	412,864.785714284	9008.92857142864	0.0218205303119824
412	117,634,330	2,706,276	417,963.357142858	8946.78571428568	0.0214056700459215
413	118,048,368	2,716,771	423,801.500000000	8922.85714285704	0.0210543311971691
414	118,507,232	2,726,927	426,103.357142858	8931.85714285728	0.0209617150231997
415	118,996,917	2,737,141	428,905	8946.92857142864	0.0208599306872819
416	119,486,174	2,747,001	436,160.857142858	8982.57142857136	0.0205946298973575
417	119,941,778	2,755,610	446,149.928571425	9022.57142857136	0.0202231825015902
418	120,306,850	2,761,528	456,514.285714284	9089.71428571432	0.0199111278007262
419	120,660,960	2,769,091	465,909.285714291	9194.35714285728	0.0197342217139143
420	121,127,990	2,779,712	474,202.857142858	9275.50000000000	0.0195601942508028
421	121,673,709	2,790,302	482,647	9476.50000000000	0.0196344326184561
422	122,221,640	2,801,022	492,823.500000000	9693.71428571432	0.0196697484712363
423	122,784,181	2,811,841	500,389.428571425	9782.28571428568	0.0195493452813609
424	123,282,611	2,820,627	509,163.214285716	9847.92857142864	0.0193413983868491
425	123,723,075	2,829,182	522,609.642857142	9913.42857142864	0.0189690885097934
426	124,144,264	2,837,149	535,592.285714284	10,112.5714285714	0.0188810998558071
427	124,650,138	2,848,606	547,277.357142858	10,353.9285714284	0.0189189785330835
428	125,279,846	2,859,279	557,154.857142858	10,364.9285714286	0.0186033172618847
429	125,932,038	2,870,833	563,605.285714284	10,324	0.0183177842040922
430	126,572,075	2,883,606	570,351.571428567	10,405.7857142857	0.0182445113427533
431	127,156,600	2,893,817	577,532.142857142	10,598.7857142857	0.0183518542567204
432	127,649,254	2,901,101	584,805.571428575	10,813.3571428570	0.0184905166283590
433	128,108,559	2,909,766	589,262.500000000	10,715	0.0181837466324431
434	128,670,765	2,921,670	586,446.571428575	10,500.9285714286	0.0179060277321573
435	129,344,669	2,934,598	588,095.142857149	10,449.0714285716	0.0177676546992154
436	130,054,493	2,946,901	595,593.642857142	10,430.0714285714	0.0175120596965020
437	130,699,295	2,957,548	601,521.642857142	10,440.2142857143	0.0173563402243098
438	131,239,632	2,966,888	603,988.357142858	10,653.5714285714	0.0176387033004537
439	131,799,554	2,974,317	612,237.214285709	10,970.4285714284	0.0179185915449904
440	132,296,570	2,982,571	628,576.714285709	11,377.6428571430	0.0181006432445912
441	132,904,057	2,995,028	645,206	11,903.8571428573	0.0184497000072183
442	133,567,214	3,010,390	665,204	12,286.7857142857	0.0184707032944566
443	134,403,269	3,024,695	684,242.642857142	12,525.6428571427	0.0183058495226786
444	135,150,593	3,039,041	703,754.857142866	12,739.2142857143	0.0181017781354057
445	135,821,218	3,052,049	725,504.142857149	12,744.2857142857	0.0175661101866306
446	136,530,824	3,061,171	735,028.571428567	12,607.5000000000	0.0171523944647439
447	137,144,697	3,071,076	742,008.071428567	12,492.2142857143	0.0168356851720810
448	137,908,498	3,084,872	758,856.642857134	12,395.0714285714	0.0163338774790233
449	138,719,831	3,098,966	766,987.642857149	12,368	0.0161254227694298
450	139,541,052	3,112,624	771,815.857142866	12,456.7857142857	0.0161395825169991
451	140,400,923	3,126,003	782,727.642857134	12,580.1428571430	0.0160721842034639
452	141,194,881	3,138,618	793,699.142857134	12,662.9285714286	0.0159543180629439
453	141,894,988	3,147,754	804,847.785714284	12,729.0714285714	0.0158155015824198
454	142,585,955	3,158,888	813,845.071428582	12,882.3571428573	0.0158290043094372
455	143,425,427	3,173,182	819,421	13,053.6428571430	0.0159303250186936
456	144,314,690	3,187,937	823,608.428571418	13,207.5000000000	0.0160361399201668
457	145,214,062	3,201,860	825,499.571428582	13,360.0714285714	0.0161842257597431
458	146,121,744	3,217,120	825,336.500000000	13,446.2142857141	0.0162917964802406
459	146,945,954	3,230,252	825,991.785714284	13,581.7857142857	0.0164430033678128
460	147,674,433	3,241,025	826,293.714285716	13,796.6428571430	0.0166970202224876
461	148,363,504	3,252,658	824,748.214285716	13,866.6428571430	0.0168131832442370
462	149,202,589	3,267,659	821,490	13,800.2857142857	0.0167990915461974
463	150,101,413	3,283,605	816,452.714285716	13,761.7857142857	0.0168555820484048
464	150,995,451	3,299,345	813,608.142857149	13,733.5714285714	0.0168798352734563
465	151,886,830	3,313,768	809,840.142857134	13,672.3571428570	0.0168827851563651
466	152,681,728	3,326,808	801,915.857142851	13,533.0714285716	0.0168759244601904
467	153,368,997	3,337,134	796,343.785714284	13,375.5714285716	0.0167962275445828
468	154,059,454	3,348,819	790,790.428571433	13,295.5000000000	0.0168129247897175
469	154,844,401	3,362,911	786,279.785714284	13,319.2142857141	0.0169395354270929
470	155,686,423	3,377,816	783,103.071428567	13,350.4285714284	0.0170481116196799
471	156,559,254	3,392,392	775,770.071428582	13,318.6428571430	0.0171682865164116
472	157,394,093	3,406,858	766,916.500000000	13,281.9285714286	0.0173186110501321
473	158,182,382	3,420,187	757,368.285714284	13,232.9285714286	0.0174722507147873
474	158,831,786	3,430,661	742,178	13,097.1428571430	0.0176469025720824
475	159,457,446	3,441,753	723,363.928571433	12,913.2857142857	0.0178517136454233
476	160,183,240	3,455,924	703,224.928571433	12,735.9285714284	0.0181107467240970
477	160,950,740	3,470,064	684,691.071428567	12,649	0.0184740250425767
478	161,685,429	3,483,504	671,483.785714284	12,653.7142857143	0.0188444077949761
479	162,395,013	3,496,532	658,034.857142851	12,664	0.0192451811063419
480	163,026,611	3,508,816	643,635.214285716	12,608.5000000000	0.0195895123824020
481	163,573,232	3,519,118	604,504.928571433	12,541.0714285714	0.0207460201494278
482	164,116,773	3,530,448	566,255.428571433	12,518.8571428573	0.0221081450370202
483	164,736,401	3,544,525	556,087.785714284	12,455.3571428573	0.0223981850758664
484	165,408,472	3,557,982	547,212.642857134	12,364	0.0225945072018886
485	165,690,766	3,571,161	535,965.785714284	12,176.8571428570	0.0227194673007511
486	166,317,252	3,584,139	523,202.642857149	11,943	0.0228267195570356
487	166,889,601	3,595,584	509,641.714285716	11,818.3571428570	0.0231895404390533
488	167,371,219	3,605,446	521,139.571428567	11,754.7857142857	0.0225559262023857
489	167,822,307	3,614,596	531,398.500000000	11,669.3571428573	0.0219597103545781
490	168,355,704	3,627,579	516,191.928571433	11,555.5000000000	0.0223860532495732
491	168,924,153	3,640,385	503,077.785714284	11,405.5714285714	0.0226715862883459
492	169,471,039	3,653,325	491,955.857142851	11,268.7142857143	0.0229059459748280
493	169,976,558	3,665,346	482,139.357142866	11,395.6428571430	0.0236355789842028
494	170,456,982	3,676,154	471,123.428571433	11,491.7857142857	0.0243923036244063
495	170,846,927	3,684,554	460,609	11,230	0.0243807654648520
496	171,233,981	3,693,250	450,110.428571433	10,934.2142857143	0.0242922927167395
497	171,693,981	3,708,464	438,369.142857134	10,737.2142857143	0.0244935449054032
498	172,181,604	3,720,385	427,602.071428567	10,559.2857142857	0.0246941874696920
499	172,662,114	3,730,545	418,155.500000000	10,437	0.0249596143061612
500	173,087,029	3,741,205	407,071.142857149	10,067.2142857143	0.0247308473281958
501	173,483,679	3,750,616	395,698.071428582	9617.42857142841	0.0243049670085749
502	173,806,659	3,757,922	388,816.571428567	9909.07142857136	0.0254852085963415
503	174,128,426	3,766,000	386,681.857142851	10,402	0.0269006673260008
504	174,498,532	3,776,655	384,456.857142851	10,507.5714285714	0.0273309507513014
505	174,916,826	3,786,838	380,995.428571433	10,676.3571428573	0.0280222709833789
506	175,370,324	3,802,819	378,662.500000000	10,810.7142857143	0.0285497356767948
507	175,792,365	3,814,559	378,103.642857134	10,811.5714285714	0.0285942006452884
508	176,160,739	3,824,368	376,355.428571433	10,811.2142857143	0.0287260750475991
509	176,463,535	3,833,639	370,167.214285716	10,326.7142857143	0.0278974309100848
510	176,772,825	3,841,633	364,735.285714284	9667.57142857136	0.0265057201955077
511	177,147,584	3,852,384	361,984.214285716	9369.35714285704	0.0258833307450853
512	177,536,750	3,862,466	360,618.428571433	9074.21428571432	0.0251629244840904
513	177,932,741	3,871,765	359,776.714285716	8823.21428571432	0.0245241393769230
514	178,336,242	3,880,959	361,406.071428567	8625.07142857136	0.0238653196789920
515	178,684,641	3,889,139	364,470.571428567	8446.57142857136	0.0231749065376237
516	178,988,291	3,895,907	365,948.857142866	8348.28571428591	0.0228127115342425
517	179,284,943	3,902,890	367,905.785714284	8305.42857142864	0.0225748789334850
518	179,695,151	3,911,878	369,951.214285702	8262.78571428568	0.0223347981982959
519	180,091,771	3,921,224	371,525.357142866	8169.14285714296	0.0219881165580890
520	180,501,004	3,929,883	374,661.357142866	8090.85714285704	0.0215951204697415
521	180,918,660	3,939,117	375,184.500000000	8001.57142857136	0.0213270309103158
522	181,281,540	3,946,660	373,312.500000000	7932.42857142864	0.0212487622874365
523	181,592,747	3,952,754	375,388.642857134	7914	0.0210821508604135
524	181,925,746	3,959,315	379,023.571428567	7844.07142857136	0.0206954712579128
525	182,306,931	3,967,475	381,607.214285716	7737.85714285704	0.0202770200698134
526	182,706,366	3,976,681	383,645.785714284	7732.64285714296	0.0201556830417050
527	183,141,850	3,985,222	387,977.571428567	7829.85714285728	0.0201812107695480
528	183,584,144	3,993,595	396,042	7910.71428571432	0.0199744327261107
529	183,958,557	4,000,512	405,685.214285716	7887.28571428568	0.0194418860647231
530	184,286,771	4,007,159	413,686.642857149	7867.78571428568	0.0190187086050117
531	184,663,408	4,014,528	422,050.500000000	7897.78571428568	0.0187128926853201
532	185,113,857	4,023,012	430,498.285714284	7878	0.0182997244389227
533	185,579,033	4,031,566	436,906.214285716	7926.14285714296	0.0181415200745111
534	186,060,796	4,040,486	443,902.642857149	8049.50000000000	0.0181334806843905
535	186,573,905	4,048,900	453,241.642857149	8094.78571428568	0.0178597572439675
536	186,995,772	4,055,499	463,788.428571418	8090.42857142841	0.0174442225657697
537	187,366,243	4,063,138	475,721.071428567	8073	0.0169700282053035
538	187,798,573	4,071,242	488,364.071428582	8077.50000000000	0.0165399145280515
539	188,324,075	4,079,625	499,143.928571433	8168.92857142864	0.0163658778637424
540	188,861,853	4,088,219	507,640.785714284	8161.71428571432	0.0160777355078557
541	189,438,071	4,096,855	515,112.285714284	8023.35714285704	0.0155759382281698
542	190,033,727	4,105,616	519,444.642857134	8838.78571428568	0.0170158376562884
543	190,523,965	4,113,148	521,207.357142851	9723.85714285704	0.0186564080679160
544	190,945,021	4,119,753	521,689.857142866	9739.28571428591	0.0186687273692170
545	191,431,367	4,126,954	530,332	9718.85714285728	0.0183259866326325
546	191,963,506	4,147,656	535,735.142857134	9771	0.0182384899147929
547	192,519,325	4,156,322	533,530.571428582	9909.50000000000	0.0185734436425383
548	193,084,257	4,165,102	539,001.785714284	10,056.0714285714	0.0186568425097982
549	193,812,189	4,173,433	547,918.785714284	9367.57142857136	0.0170966421900638
550	194,245,795	4,182,125	559,928.214285716	8692.35714285704	0.0155240563363031
551	194,692,619	4,189,509	572,209.642857134	8902.64285714296	0.0155583586684954
552	195,229,794	4,197,983	578,077.500000000	9099.57142857136	0.0157410925499978
553	195,835,942	4,207,773	584,526.071428582	9202.71428571409	0.0157438901967583
554	196,485,884	4,217,898	593,395.642857149	9217.42857142864	0.0155333607221103
555	197,128,633	4,228,163	598,225.214285702	9193.14285714319	0.0153673610500020
556	197,860,898	4,237,766	602,321.571428567	9206.57142857136	0.0152851431283384
557	198,380,451	4,246,630	606,500.500000000	9272.64285714272	0.0152887637473386
558	198,865,502	4,254,048	611,736.571428567	9350.35714285728	0.0152849405766631
559	199,432,064	4,262,148	621,013.357142866	9464.21428571409	0.0152399528558559
560	200,066,174	4,272,500	630,436.785714299	9514.21428571409	0.0150914643645584
561	200,746,659	4,282,988	632,617.428571433	9507.71428571455	0.0150291690622320
562	201,432,170	4,293,978	634,894.142857134	9563.14285714319	0.0150625784860881
563	202,251,548	4,304,450	639,011.357142851	9697	0.0151750041554148
564	202,815,916	4,313,145	643,420.714285716	9783.50000000000	0.0152054476686549
565	203,286,681	4,320,641	648,447.928571433	9751.92857142864	0.0150388768962724
566	203,899,403	4,329,439	648,636.428571433	9717.78571428545	0.0149818685572256
567	204,544,994	4,340,967	646,962.928571418	9721.14285714272	0.0150258112603273
568	205,275,729	4,351,490	646,579	9721.28571428591	0.0150349542968236
569	205,981,371	4,362,003	649,563	9792.71428571409	0.0150758498955668
570	206,783,257	4,372,474	655,145.428571418	9808.78571428545	0.0149719211743171
571	207,341,688	4,381,217	658,137.214285716	9771.35714285728	0.0148469907653866
572	207,813,015	4,388,667	658,914.357142866	9859.71428571455	0.0149635748239991
573	208,466,951	4,398,511	658,154.071428582	9928.92857142864	0.0150860246900502
574	209,149,482	4,409,218	657,582.642857134	9944.85714285728	0.0151233571185027
575	209,885,162	4,420,038	657,031.857142851	9982.50000000000	0.0151933272207068
576	210,596,739	4,431,491	656,938.142857149	10,019.8571428568	0.0152523601374074
577	211,382,046	4,441,991	657,590.428571433	10,056	0.0152921933822028
578	211,949,056	4,450,928	657,296.857142851	10,114	0.0153872635934450
579	212,404,093	4,458,711	659,191.571428567	10,159.5714285714	0.0154121682814512
580	213,073,007	4,468,745	658,518.428571433	10,163.3571428573	0.0154336715601191
581	213,749,692	4,479,768	656,107	10,122.5714285714	0.0154282326336579
582	214,487,108	4,491,084	656,147.785714284	10,020.5000000000	0.0152717119803912
583	215,223,475	4,502,679	655,027.071428567	9917.21428571455	0.0151401594198020
584	215,974,568	4,513,090	649,884.785714284	9759.71428571409	0.0150176069670369
585	216,542,032	4,521,545	644,574.571428582	9716.85714285681	0.0150748378443183
586	216,997,186	4,528,381	639,009.285714299	9768.14285714319	0.0152863864039537
587	217,650,293	4,537,916	632,781	9762.92857142864	0.0154286057442127
588	218,270,793	4,547,233	626,531.928571418	9738	0.0155427035014864
589	218,990,051	4,559,655	622,397.357142851	9673.57142857136	0.0155424365440406
590	219,666,662	4,570,862	608,500.857142866	9554.92857142864	0.0157024077439965
591	220,390,315	4,581,588	597,014.071428567	9535.35714285728	0.0159717460595871
592	220,897,732	4,589,379	593,760	9451.35714285681	0.0159178070985867
593	221,355,049	4,595,977	585,331.357142866	9252.64285714272	0.0158075297764790
594	221,811,442	4,604,089	578,089.571428567	9109.92857142864	0.0157586800068306
595	222,467,841	4,614,555	571,468.785714284	9044.35714285728	0.0158265112092739
596	223,105,643	4,624,652	563,861.071428567	9022.71428571455	0.0160016620102095
597	223,745,709	4,635,402	565,596.785714284	9027.71428571409	0.0159613960222796
598	224,404,522	4,644,587	565,331.785714284	9059.14285714272	0.0160244710912490
599	224,884,088	4,653,001	552,656.642857149	9043.42857142864	0.0163635571711859
600	225,262,748	4,658,673	543,830.428571433	9051	0.0166430554902485
601	225,822,098	4,667,781	537,521.285714284	9025.14285714319	0.0167902985370154
602	226,371,830	4,677,691	538,775.428571433	8882.78571428545	0.0164869911343920
603	226,938,847	4,688,124	541,999.071428567	8782.57142857136	0.0162040340870378
604	227,526,131	4,698,644	538,187.571428567	8755.50000000000	0.0162684916278527
605	228,149,398	4,707,697	530,579.142857149	8656.42857142864	0.0163150562700488
606	228,682,068	4,714,250	523,929.071428567	8566.42857142864	0.0163503593111775
607	229,052,755	4,720,380	518,808	8467.71428571409	0.0163214797877328
608	229,566,717	4,728,651	510,686.357142866	8394.35714285728	0.0164374023810253
609	230,055,319	4,738,011	494,576.500000000	8296.64285714272	0.0167752468165041
610	230,590,365	4,747,734	483,240.071428567	8183.50000000000	0.0169346469464085
611	231,137,925	4,757,582	479,431.785714284	8101.21428571455	0.0168975327191645
612	231,687,213	4,766,280	473,439.642857149	8060.85714285728	0.0170261558457822
613	232,068,324	4,771,820	467,603.642857134	8031.42857142864	0.0171757185687333
614	232,431,860	4,777,379	460,337.285714284	7931.57142857136	0.0172299130978807
615	232,899,657	4,785,069	454,266.142857149	7857.71428571409	0.0172976005570044
616	233,350,534	4,794,445	450,747.142857134	7884.71428571409	0.0174925441251508
617	233,841,601	4,803,740	445,720.428571433	7831.28571428591	0.0175699501577385
618	234,331,411	4,812,618	439,738.285714299	7708.78571428591	0.0175303947023034
619	234,853,453	4,821,252	435,200.642857134	7528.14285714272	0.0172980968220077
620	235,212,544	4,827,234	434,337.928571418	7373.57142857136	0.0169765773226939
621	235,527,726	4,831,603	433,479	7368.07142857136	0.0169975279738381
622	235,960,127	4,838,768	428,146.357142866	7297.28571428591	0.0170439047128245
623	236,382,873	4,846,140	424,095.357142866	7142.35714285728	0.0168413943292740
624	236,889,993	4,855,275	424,001.357142851	7093.64285714272	0.0167302362071280
625	237,351,725	4,864,236	420,281.785714284	7030.92857142864	0.0167290822738828
626	237,827,188	4,871,796	417,029.357142866	6982	0.0167422266092603
627	238,176,144	4,876,683	414,507.857142851	7008.92857142817	0.0169090367061793
628	238,500,145	4,881,465	410,285.071428567	6924.57142857136	0.0168774637703994
629	238,871,653	4,887,339	408,435.642857149	6831.28571428591	0.0167254886632780
630	239,309,758	4,895,317	407,423.071428567	6843.92857142864	0.0167980879124774
631	239,766,218	4,904,223	407,506.357142851	6852.92857142864	0.0168167402822292
632	240,219,491	4,912,232	409,972.857142866	6869.21428571409	0.0167552904199224
633	240,677,521	4,919,438	412,115.785714284	6915.07142857136	0.0167794383721218
634	241,029,734	4,924,856	413,062	6898.78571428591	0.0167015743745150
635	241,351,644	4,929,233	415,099.785714284	6864.07142857136	0.0165359551240433
636	241,759,774	4,935,740	418,203.285714284	6915.14285714272	0.0165353623306230
637	242,191,258	4,943,727	423,050.714285716	7023.57142857136	0.0166021973049497
638	242,667,586	4,952,396	425,481.642857134	7083.57142857136	0.0166483596824642
639	243,129,520	4,960,156	426,646	7124.42857142864	0.0166986883070007
640	243,622,338	4,968,326	427,375.142857149	7127.42857142864	0.0166772183421322
641	244,007,627	4,974,298	429,699.214285716	7143.92857142864	0.0166254168821414
642	244,330,494	4,978,961	433,889.500000000	7261.57142857136	0.0167359925247589
643	244,753,968	4,985,754	435,482.142857149	7320.28571428591	0.0168096116783534
644	245,180,316	4,993,497	436,340.071428567	7303.78571428591	0.0167387462040181
645	245,694,317	5,002,641	440,158.714285716	7312.64285714272	0.0166136500762221
646	246,177,242	5,011,573	442,904.142857149	7282.57142857136	0.0164427710736502
647	246,671,366	5,019,393	441,020	7203.14285714272	0.0163329165505935
648	247,067,360	5,025,484	440,213.714285716	7105.64285714272	0.0161413482282628
649	247,432,983	5,030,152	443,444.428571418	6959.85714285728	0.0156949928659130
650	247,852,137	5,036,519	448,056.285714284	6917.21428571455	0.0154382708294947
651	248,256,427	5,043,576	453,321.500000000	7021.42857142864	0.0154888496826836
652	248,781,198	5,052,041	456,329.500000000	7069.85714285681	0.0154928777185275
653	249,298,583	5,059,611	459,668.928571433	7088.50000000000	0.0154208813330711
654	249,822,813	5,068,196	467,753.785714284	7176	0.0153414044293450
655	250,262,414	5,074,981	475,873.285714284	7229.78571428545	0.0151926698373782
656	250,626,542	5,079,633	479,585.357142851	7223.28571428591	0.0150615226397214
657	251,093,943	5,086,277	485,200.428571433	7260.42857142864	0.0149637719669898
658	251,563,174	5,094,282	490,655.214285716	7271	0.0148189600116345
659	252,136,677	5,102,552	491,970.428571433	7261.07142857136	0.0147591623538346
660	252,657,299	5,110,226	497,178.285714284	7284.35714285728	0.0146513984060909
661	253,256,903	5,119,227	503,817.357142851	7270.57142857136	0.0144309665506619
662	253,697,497	5,125,744	509,468.714285716	7306.35714285681	0.0143411301577182
663	254,079,045	5,130,525	519,645.500000000	7414	0.0142674188461172
664	254,601,936	5,137,366	527,910.714285716	7385.14285714319	0.0139893786151614
665	255,108,624	5,144,981	533,339.571428567	7295.07142857183	0.0136780989436650
666	255,723,789	5,154,142	540,610.714285716	7272.92857142864	0.0134531713472198
667	256,345,224	5,162,432	548,876.928571433	7288.78571428545	0.0132794536167808
668	256,959,728	5,170,413	560,352.714285716	7338.42857142817	0.0130960882036281
669	257,461,426	5,176,689	569,480.857142851	7325.07142857136	0.0128627175728471
670	257,883,666	5,181,401	570,772.571428567	7251.71428571455	0.0127050854380835
671	258,481,592	5,188,533	568,972.357142866	7143	0.0125542127140747
672	259,073,906	5,196,552	567,746.214285716	7057.71428571409	0.0124311076113355
673	259,731,239	5,205,122	567,438.500000000	7071	0.0124612623218199
674	260,328,590	5,212,976	570,614.857142851	7119.78571428591	0.0124773928073582
675	260,941,975	5,219,871	573,509.142857134	7148.35714285728	0.0124642426923575
676	261,427,626	5,226,039	577,263.428571433	7197.50000000000	0.0124683110756069
677	261,861,605	5,231,045	588,314.785714299	7406.78571428591	0.0125898343780244
678	262,492,261	5,238,566	604,535	7688.85714285728	0.0127186302577308
679	263,092,365	5,246,596	616,539.857142851	7790.42857142817	0.0126357257866999
680	263,794,468	5,255,843	622,142.642857149	7949.85714285681	0.0127781903943244
681	264,501,768	5,265,950	621,713.142857149	8090.92857142864	0.0130139255770691
682	265,232,287	5,274,541	622,095	8031.42857142864	0.0129102927550111
683	265,768,872	5,280,435	625,516	7941.92857142864	0.0126966033985200
684	266,230,356	5,287,947	625,498.642857134	7764.21428571455	0.0124128395391066
685	266,827,494	5,294,937	622,183.285714284	7613.14285714272	0.0122361738605733
686	267,466,462	5,302,665	618,368.142857149	7604.07142857136	0.0122969973735015
687	268,177,595	5,310,961	617,027.142857134	7437.14285714319	0.0120531858982826
688	268,875,622	5,319,531	617,589.142857134	7188.42857142864	0.0116394995840973
689	269,568,999	5,327,544	618,153.285714299	7139.64285714272	0.0115499553624351
690	270,089,314	5,333,889	620,045.571428567	7192.78571428545	0.0116004146239017
691	270,548,294	5,338,613	625,387.428571433	7157.71428571409	0.0114452481113418
692	271,155,804	5,344,909	633,949.785714299	7019.35714285728	0.0110724181962587
693	271,792,298	5,352,648	642,960.571428567	6949.64285714319	0.0108088165370733
694	272,532,397	5,361,677	650,616.500000000	6916.64285714272	0.0106309060055236
695	273,276,244	5,369,023	663,856.857142836	6930.57142857136	0.0104398581621944
696	274,043,674	5,376,323	681,863.428571433	6945.78571428591	0.0101864763869768
697	274,616,087	5,382,405	703,010.357142866	7028.78571428545	0.00999812540863756
698	275,130,152	5,386,930	734,573.642857134	7118.42857142864	0.00969055810897518
699	275,867,942	5,393,620	762,525.142857134	7023.78571428591	0.00921121851532426
700	276,626,248	5,401,178	780,742.642857134	6820.21428571409	0.00873554729988291
701	277,540,592	5,411,550	795,232.428571433	6661.57142857136	0.00837688603888841
702	278,552,080	5,418,808	838,624.714285731	6594.14285714272	0.00786304379636492
703	279,443,190	5,424,871	918,166.642857164	6579.14285714272	0.00716552154047946
704	280,146,968	5,429,340	1,015,861.35714284	6415.28571428591	0.00631511935086225
705	280,732,525	5,433,257	1,139,244.85714284	6254.14285714319	0.00548972665352051
706	282,006,315	5,439,611	1,267,064.92857143	6310.07142857136	0.00498006951836772
707	283,342,208	5,447,295	1,365,436.35714287	6353.28571428545	0.00465293433930491
708	285,046,691	5,455,247	1,427,574.42857143	6315.21428571409	0.00442373732627986
709	286,995,409	5,462,669	1,529,568.85714287	6244.35714285728	0.00408242957726096
710	288,738,770	5,469,351	1,691,036.78571430	6238.64285714319	0.00368924136355081
Year 2022					
711	289,967,497	5,473,806	1,837,958.78571427	6254.14285714272	0.00340276556022568
712	290,898,038	5,477,204	1,952,098.85714284	6255.57142857136	0.00320453618713104
713	293,254,766	5,483,085	2,089,859.42857143	6310.57142857136	0.00301961526325485
714	295,768,272	5,491,162	2,245,004.78571430	6424.21428571409	0.00286155928334473
715	298,352,050	5,498,938	2,393,040.14285713	6549.42857142864	0.00273686531794199
716	301,019,434	5,506,556	2,529,693.57142857	6651.28571428591	0.00262928513927867
717	303,972,777	5,513,812	2,623,843.21428573	6774.07142857136	0.00258173635973726
718	306,163,557	5,519,284	2,722,294.92857143	6936.71428571409	0.00254811270186447
719	308,204,540	5,523,418	2,820,945.57142857	7069.50000000000	0.00250607458421110
720	311,363,974	5,529,989	2,883,904.92857143	7163.92857142864	0.00248410705236991
721	314,392,869	5,539,095	2,932,311.78571430	7278.42857142864	0.00248214688727435
722	317,839,582	5,548,119	2,967,798.64285713	7372.28571428591	0.00248409228571805
723	321,025,140	5,556,348	2,944,159.35714284	7377.64285714319	0.00250585717761651
724	324,341,740	5,564,315	2,960,019.21428573	7352.85714285681	0.00248405723428086
725	326,846,959	5,570,679	3,058,062.35714287	7417.64285714272	0.00242560222482611
726	329,070,319	5,575,235	3,142,631.21428570	7583.21428571455	0.00241301437191960
727	331,716,426	5,581,459	3,218,432.85714287	7826.78571428545	0.00243186235714534
728	335,480,686	5,590,565	3,275,030.42857143	7957.85714285728	0.00242985746740939
729	339,564,638	5,600,496	3,312,318.57142857	7993.35714285728	0.00241322112305455
730	343,296,921	5,610,136	3,384,575.57142857	8216.21428571409	0.00242754641233972
731	347,128,019	5,620,102	3,431,487.78571427	8490.14285714272	0.00247418711279938
732	349,911,106	5,626,302	3,400,561.21428570	8644.50000000000	0.00254208039651943
733	352,378,632	5,631,519	3,375,154.71428573	8747.85714285728	0.00259183885877319
734	355,792,171	5,640,202	3,361,902.85714287	8965.21428571409	0.00266670830974968
735	359,445,770	5,650,684	3,348,758.78571427	9269	0.00276789120779357
736	363,207,411	5,661,400	3,334,159.35714287	9430.57142857183	0.00282847051337497
737	366,906,314	5,671,702	3,331,744.21428573	9566.42857142864	0.00287129742145572
738	370,585,266	5,684,049	3,304,194.42857143	9831.85714285681	0.00297556858574682
739	373,336,482	5,692,121	3,225,532.14285713	10,072.9285714286	0.00312287341291418
740	375,631,487	5,697,728	3,148,204.42857143	10,251.9285714286	0.00325643674165107
741	379,183,735	5,707,923	3,060,400.85714287	10,405.5714285714	0.00340006813299805
742	382,312,928	5,720,609	2,977,958	10,486.0714285714	0.00352122878447962
743	385,497,703	5,732,496	2,918,653.50000000	10,537.4285714286	0.00361037326679191
744	388,690,884	5,744,133	2,823,779.28571427	10,617.6428571432	0.00376008242246790
745	391,646,308	5,757,296	2,719,597.71428570	10,676.5000000000	0.00392576444079126
746	393,966,852	5,765,679	2,628,039.07142857	10,772.9285714282	0.00409922694397848
747	395,862,266	5,771,694	2,547,237.50000000	10,878.7142857141	0.00427078915323525
748	398,485,866	5,782,604	2,482,238.50000000	10,811.4285714286	0.00435551562487998
749	401,085,165	5,795,399	2,416,747.71428573	10,759.9285714286	0.00445223492209187
750	403,518,013	5,808,527	2,363,107.57142857	10,743.8571428573	0.00454649516287670
751	406,331,899	5,820,404	2,271,659.50000000	10,699.7142857146	0.00471008717887278
752	408,756,632	5,832,385	2,154,219.57142860	10,551.5714285714	0.00489809468288043
753	410,690,996	5,841,229	2,088,367.64285713	10,397.2857142854	0.00497866635208958
754	412,221,628	5,846,558	2,020,855.64285713	10,420	0.00515623173621049
755	413,929,737	5,857,536	1,935,610.14285713	10,348.1428571427	0.00534619168809889
756	415,800,368	5,868,189	1,884,904	10,232.4285714286	0.00542862054058384
757	418,039,957	5,881,299	1,850,085.28571430	10,221.2142857146	0.00552472600297896
758	420,101,934	5,893,512	1,812,311.14285713	9965.57142857136	0.00549881926613357
759	422,085,139	5,904,151	1,776,088.35714287	9658.35714285728	0.00543799361333259
760	423,751,145	5,912,717	1,738,945.42857143	9535.14285714272	0.00548329044746158
761	425,062,673	5,918,167	1,696,307.78571427	9354.42857142817	0.00551458211193040
762	426,461,048	5,925,445	1,653,204.21428570	9141.64285714319	0.00552965131479114
763	428,134,294	5,935,497	1,614,243.71428573	8902.35714285728	0.00551487799771076
764	430,051,267	5,947,483	1,587,972.92857143	8725.78571428545	0.00549492095065834
765	431,838,933	5,958,290	1,571,918	8682.92857142864	0.00552377959373748
766	433,492,999	5,967,356	1,561,313.50000000	8577.07142857136	0.00549349725636226
767	434,942,697	5,974,145	1,537,491.78571427	8192.07142857136	0.00532820500550876
768	436,102,742	5,978,900	1,518,125.57142857	7731.64285714319	0.00509288757310613
769	437,427,831	5,986,273	1,524,932.14285713	7518.57142857136	0.00493043015965580
770	439,025,900	5,994,748	1,534,994.57142860	7429.78571428545	0.00484026839741241
771	440,684,546	6,002,921	1,542,091	7331.85714285728	0.00475449058639035
772	442,459,412	6,011,095	1,554,500.92857140	7310.92857142864	0.00470307121536906
773	444,221,570	6,019,811	1,572,331.57142860	7233.85714285728	0.00460071989541284
774	445,704,050	6,025,707	1,594,890.35714287	7122.07142857136	0.00446555551400414
775	446,930,663	6,029,984	1,614,124.07142857	7054.92857142864	0.00437074738943998
776	448,362,923	6,037,542	1,628,845.14285713	6830.21428571455	0.00419328646167908
777	450,103,450	6,044,753	1,659,234.50000000	6585.64285714272	0.00396908505527261
778	451,935,461	6,052,625	1,685,868.28571430	6471.78571428545	0.00383884421406229
779	453,806,234	6,060,160	1,702,974.28571427	6263.21428571409	0.00367780907689228
780	455,678,580	6,066,369	1,720,825.21428570	5989.21428571455	0.00348043150227818
781	457,476,323	6,071,348	1,754,561.35714287	5805.21428571455	0.00330864136616333
782	458,760,546	6,074,948	1,798,208.21428573	5702.85714285681	0.00317141090645170
783	460,374,680	6,080,263	1,814,332.85714287	5622	0.00309865964112742
784	462,183,246	6,085,881	1,811,533.64285713	5508.71428571455	0.00304091194079413
785	464,419,524	6,092,770	1,793,693.71428570	5427.85714285681	0.00302607803084059
786	466,497,086	6,099,855	1,770,994.57142857	6181.50000000000	0.00349041160245551
787	468,388,388	6,105,382	1,773,971.71428573	6912.71428571455	0.00389674436748157
788	470,127,986	6,109,457	1,755,050.71428573	6809.21428571409	0.00387978206571956
789	471,220,595	6,112,829	1,702,958.78571427	6614.71428571409	0.00388424801657175
790	472,708,555	6,128,923	1,672,343.78571427	6729.42857142864	0.00402395047532315
791	474,684,975	6,133,999	1,636,969.28571430	6904	0.00421755011547905
792	476,488,505	6,139,981	1,601,697	6785	0.00423613205244188
793	478,269,528	6,145,250	1,591,851	5868.64285714319	0.00368667850015057
794	480,028,759	6,154,199	1,575,262.21428573	4972.50000000000	0.00315661732688400
795	481,405,185	6,157,296	1,544,356.14285713	4868.28571428545	0.00315230766996457
796	482,367,154	6,159,980	1,531,990.71428570	4760.28571428591	0.00310725493953494
797	483,847,910	6,163,933	1,492,097.07142857	4414	0.00295825257251791
798	485,599,291	6,168,604	1,427,782.57142857	4083.78571428545	0.00286022941868482
799	487,195,175	6,173,532	1,394,851.85714287	4015.28571428591	0.00287864671342991
800	489,010,728	6,178,343	1,343,931.28571430	3897.42857142864	0.00290002071747080
801	490,176,918	6,182,902	1,276,389.92857143	3795.21428571409	0.00297339723603255
802	491,245,982	6,185,766	1,236,335.71428570	3740.78571428591	0.00302570383679903
803	492,054,283	6,187,724	1,183,603	3686.35714285728	0.00311452162833085
804	492,975,819	6,190,753	1,142,145	3604.92857142864	0.00315627925651177
805	494,340,841	6,194,917	1,121,581.21428570	3523.42857142864	0.00314148322613677
806	495,762,325	6,199,590	1,086,779	3474.71428571409	0.00319725931924898
807	497,014,020	6,203,894	1,073,370.64285713	3445.42857142864	0.00320991504132951
808	498,163,656	6,207,820	1,056,107.85714287	3369.92857142864	0.00319089432829849
809	498,961,381	6,210,176	1,011,192.92857143	3265.35714285681	0.00322921279470373
810	499,553,790	6,211,960	964,280.785714299	3158.21428571409	0.00327520192510589
811	500,503,501	6,214,753	910,625.571428567	2995.21428571455	0.00328918315023345
812	501,598,669	6,218,096	863,707.428571403	2841.07142857136	0.00328939098424866
813	502,661,198	6,222,126	837,445.857142866	2771.64285714272	0.00330963826915188
814	503,615,078	6,225,573	792,152.357142866	2668.42857142864	0.00336857998005980
815	504,311,356	6,228,074	747,458.857142866	2563.28571428591	0.00342933352088967
816	504,905,585	6,229,697	728,188.642857134	2554.21428571455	0.00350762719354258
817	505,333,828	6,231,242	715,978.500000000	2535.21428571409	0.00354090840117976
818	505,813,596	6,232,829	719,679.928571433	2522	0.00350433560792251
819	506,752,998	6,235,906	721,968.785714269	2536.92857142864	0.00351390340084962
820	507,701,510	6,240,075	713,431.714285731	2509.50000000000	0.00351750552961112
821	508,598,465	6,243,117	717,521.642857164	2525.21428571409	0.00351935626033343
822	509,403,488	6,245,838	703,444.571428567	2631.35714285681	0.00374067446069419
823	509,921,016	6,247,450	672,615.285714269	2639.64285714319	0.00392444672787964
824	510,306,441	6,248,622	651,148.285714269	2577.64285714319	0.00395861113926711
825	510,886,286	6,250,802	626,788.428571433	2555.21428571409	0.00407667750270677
826	511,528,532	6,254,772	603,397.285714299	2528.21428571409	0.00418996628849797
827	512,342,590	6,258,164	587,361.357142866	2519.14285714272	0.00428891486732585
828	513,073,461	6,261,115	573,509.571428567	2494.85714285728	0.00435015781278555
829	513,703,530	6,263,613	566,455.142857134	2341.35714285728	0.00413334960831628
830	514,068,536	6,265,070	554,019.785714299	2234.35714285728	0.00403299160151206
831	514,381,980	6,266,270	529,770.571428567	2205.92857142864	0.00416393187994603
832	514,839,881	6,268,082	518,740	2130.50000000000	0.00410706712418553
833	515,505,309	6,270,271	516,613.857142866	2081	0.00402815366105156
834	516,122,090	6,273,946	513,127.714285702	2033	0.00396197660621398
835	516,710,749	6,276,216	529,183.928571433	2014.64285714272	0.00380707490981706
836	517,328,602	6,278,339	545,006.928571433	2008.85714285681	0.00368592954978097
837	517,676,058	6,279,478	547,519	1890.92857142864	0.00345363096336134
838	517,958,246	6,280,324	555,650.785714299	1762.92857142864	0.00317272757773997
839	518,672,190	6,282,233	555,088.571428567	1721.92857142864	0.00310207894750402
840	519,303,097	6,284,244	558,304.785714269	1691.28571428591	0.00302932333299304
841	519,989,568	6,286,446	571,934.357142866	1673.92857142864	0.00292678442993153
842	520,622,382	6,288,397	570,934.142857134	1650.21428571409	0.00289037589774522
843	521,188,209	6,290,265	560,622.714285702	1617.28571428545	0.00288480233332333
844	521,632,718	6,291,230	562,337.214285731	1615.28571428591	0.00287245032562466
845	522,008,667	6,292,007	563,335	1609.21428571455	0.00285658495515910
846	522,614,847	6,293,653	563,829.285714269	1599.28571428545	0.00283647152570205
847	523,209,158	6,295,466	562,398.928571433	1615.42857142864	0.00287238913404768
848	523,956,228	6,297,838	552,950.428571433	1611.42857142864	0.00291423695174958
849	524,542,412	6,299,534	541,886.642857134	1596.28571428545	0.00294579269544073
850	525,161,789	6,301,518	533,944	1581.35714285728	0.00296165354954317
851	525,532,723	6,302,593	524,816.500000000	1586	0.00302200864492637
852	525,849,968	6,303,204	510,917.714285731	1582.85714285728	0.00309806667218444
853	526,359,959	6,304,804	501,409.928571433	1558.50000000000	0.00310823522071117
854	526,939,262	6,306,454	491,837.500000000	1530.64285714272	0.00311209059321976
855	527,573,555	6,309,054	483,082.142857134	1508.28571428591	0.00312221376133949
856	528,077,933	6,310,478	472,820.142857134	1483.50000000000	0.00313755668494912
857	528,646,007	6,312,393	468,211.071428597	1445.78571428545	0.00308789305189664
858	528,934,230	6,313,147	475,143.071428567	1395.14285714272	0.00293625844726743
859	529,211,611	6,313,766	480,714.714285702	1371.85714285728	0.00285378646022045
860	529,617,798	6,315,011	480,294.142857134	1360	0.00283159813673709
861	530,236,378	6,316,488	475,911.928571433	1326.64285714272	0.00278758059526890
862	530,928,442	6,318,552	472,601.357142866	1334	0.00282267492430568
863	531,453,052	6,320,186	473,978.428571433	1328.35714285728	0.00280256877272017
864	531,995,006	6,321,725	478,419	1351.78571428591	0.00282552681704931
865	532,247,998	6,322,388	481,234.500000000	1428.64285714272	0.00296870414972892
866	532,514,262	6,323,201	484,663.857142866	1456.42857142864	0.00300502822722207
867	532,950,845	6,324,173	487,684.785714269	1460.14285714319	0.00299402995523973
868	533,601,197	6,326,251	490,010.928571433	1487.92857142817	0.00303652119712115
869	534,300,906	6,328,790	487,717.285714299	1488.78571428545	0.00305255884483366
870	534,865,882	6,330,338	492,274.142857134	1526.35714285728	0.00310062424566599
871	535,409,763	6,332,015	515,114.857142866	1562.71428571455	0.00303372008018231
872	535,693,394	6,332,929	530,288.071428567	1503.85714285728	0.00283592489419188
873	535,896,908	6,333,503	528,555.928571403	1481.50000000000	0.00280292003157403
874	536,460,037	6,335,240	528,403.000000030	1472.78571428545	0.00278723950145129
875	537,303,613	6,337,062	527,414.928571433	1426.50000000000	0.00270470159777966
876	538,022,523	6,339,033	528,607.500000000	1414.71428571455	0.00267630384683258
877	538,544,048	6,340,836	549,972.785714269	1417.28571428545	0.00257701062870733
878	539,129,239	6,342,136	563,979.357142806	1398.78571428545	0.00248020729228793
879	539,357,727	6,342,779	567,961.571428597	1389.71428571455	0.00244684562411326
880	539,633,080	6,343,459	592,073.142857194	1393.42857142864	0.00235347370209077
881	540,423,484	6,345,126	622,349.928571403	1427	0.00229292225239852
882	541,235,877	6,346,759	641,026.714285672	1471.28571428591	0.00229520187146247
883	542,041,721	6,348,792	648,187.642857194	1479.85714285681	0.00228306904515125
884	542,813,874	6,350,585	658,652.142857134	1466.57142857136	0.00222662515331628
885	543,572,312	6,352,365	676,919.714285672	1480.35714285728	0.00218690209727374
886	543,889,028	6,353,148	694,490.714285731	1486.28571428591	0.00214010883617721
887	544,176,406	6,353,808	723,838	1436.28571428591	0.00198426404013870
888	545,101,288	6,355,309	756,919.714285731	1414	0.00186809772993470
889	546,034,949	6,357,301	772,598	1424.78571428545	0.00184414885138901
890	546,965,519	6,359,058	780,865.428571403	1433.07142857136	0.00183523482553604
891	548,023,808	6,360,427	784,204.214285731	1413.14285714272	0.00180200875154674
892	548,959,254	6,362,319	800,479.071428597	1404.57142857136	0.00175466352426261
893	549,318,458	6,363,141	840,058.714285731	1463.07142857183	0.00174162996430055
894	549,679,092	6,363,878	859,822.571428537	1600.57142857136	0.00186151362124877
895	550,577,461	6,365,023	861,591.642857134	1726.21428571409	0.00200351790784526
896	551,765,483	6,367,251	875,316.642857194	1768.14285714272	0.00202000369988531
897	552,995,807	6,369,591	890,303.571428537	1788.57142857136	0.00200894558437130
898	554,031,036	6,372,302	910,027.142857134	1847	0.00202960979185866
899	555,014,309	6,374,611	925,638.214285731	1908.28571428591	0.00206158916608520
900	555,517,836	6,375,603	941,967.928571403	1942.78571428591	0.00206247543611422
901	555,943,964	6,376,456	952,331.428571463	1917.21428571409	0.00201317968534336
902	557,052,969	6,378,303	949,164.714285731	1870.85714285728	0.00197105635586668
903	558,248,910	6,380,687	954,914.357142806	1878.64285714272	0.00196734172346492
904	559,699,931	6,383,354	960,793.571428537	1893.35714285681	0.00197061803821367
905	560,659,552	6,385,380	972,236.285714328	1917.50000000000	0.00197225718498170
906	561,674,099	6,387,725	1,004,831.71428573	1983.78571428591	0.00197424671821396
907	562,226,847	6,388,790	1,038,811.42857140	2055.28571428591	0.00197849740362636
908	562,686,063	6,389,776	1,061,084.78571427	2106.78571428545	0.00198550176446764
909	563,922,178	6,391,828	1,077,479.71428573	2177.71428571409	0.00202111859447647
910	565,447,345	6,394,935	1,088,050.85714287	2230.57142857183	0.00205006173555998
911	567,044,856	6,397,880	1,100,582	2247.28571428591	0.00204190665873684
912	568,169,814	6,400,349	1,092,208.64285713	2248.28571428545	0.00205847639916500
913	569,248,553	6,403,244	1,052,141.21428573	2231.92857142864	0.00212132035236718
914	569,885,105	6,404,499	1,013,527.21428573	2246.35714285728	0.00221637575310729
915	570,435,953	6,405,529	998,027.714285672	2293.71428571409	0.00229824708560903
916	571,463,209	6,407,551	1,015,808.85714287	2320.14285714272	0.00228403487607749
917	572,636,291	6,410,459	1,031,302.28571433	2315	0.00224473467388519
918	574,045,291	6,413,805	1,026,590.64285713	2294.42857142864	0.00223499852389356
919	575,141,767	6,416,536	1,017,783.21428567	2320.07142857136	0.00227953398720542
920	576,497,924	6,419,539	1,009,545.21428573	2353.57142857136	0.00233131849397805
921	577,073,966	6,420,614	990,688.071428597	2341.28571428591	0.00236329252547648
922	577,619,361	6,421,536	996,105.642857134	2355.57142857136	0.00236478072929581
923	578,528,766	6,424,025	987,335.714285731	2378.57142857136	0.00240908071505556
924	579,704,367	6,426,935	997,119.357142866	2419.42857142864	0.00242641821573020
925	580,846,848	6,430,107	1,036,727.64285713	2465.28571428591	0.00237794924372981
926	582,285,689	6,433,212	1,028,784.14285713	2455.71428571455	0.00238700635382516
927	583,176,702	6,436,163	1,014,149.21428567	2494	0.00245920419290240
928	584,354,859	6,437,862	994,885.142857134	2507.21428571409	0.00252010425898392
929	584,852,655	6,438,802	954,020.857142866	2477.64285714272	0.00259705313420804
930	585,698,450	6,441,139	912,176.571428597	2475.07142857136	0.00271336877759867
931	586,732,772	6,444,737	863,328	2447.78571428591	0.00283529054343878
932	587,746,835	6,447,406	821,980.428571403	2433.21428571455	0.00296018518341545
933	588,741,994	6,450,600	821,315.857142866	2441.85714285681	0.00297310361369543
934	589,490,869	6,453,426	827,069.714285731	2438.21428571409	0.00294801543763425
935	590,127,284	6,454,868	828,532.571428597	2472.21428571455	0.00298384682867909
936	590,587,956	6,455,861	821,765.357142866	2496	0.00303736337666771
937	591,461,571	6,458,266	820,319.785714269	2467.71428571409	0.00300823450645579
938	592,548,627	6,461,745	818,898.357142866	2438.07142857136	0.00297725768687312
939	593,530,436	6,465,009	816,160.785714269	2469.85714285728	0.00302618942993661
940	594,463,108	6,467,941	807,980.428571403	2453.57142857136	0.00303667185715073
941	595,254,232	6,470,633	783,432.642857134	2346.14285714272	0.00299469632588511
942	595,828,498	6,471,794	758,043.285714269	2291.14285714319	0.00302244330940067
943	596,312,993	6,473,513	745,144	2281.14285714272	0.00306134499793694
944	597,048,260	6,474,964	736,319.714285731	2278.28571428545	0.00309415281172460
945	597,929,995	6,477,893	720,978.428571463	2270.85714285728	0.00314968805288214
946	598,761,674	6,480,937	706,689.857142866	2222.14285714272	0.00314443858884123
947	599,663,886	6,483,949	691,325.857142866	2214.21428571455	0.00320285182860877
948	600,361,930	6,486,521	668,122.857142866	2216.28571428591	0.00331718289621695
949	600,814,498	6,487,698	651,908	2208.42857142817	0.00338763839595184
950	601,220,651	6,488,719	632,834.285714269	2209	0.00349064526032552
951	601,819,164	6,490,757	613,318.714285672	2187.92857142864	0.00356735987418369
952	602,512,811	6,493,128	604,669.214285731	2188.78571428591	0.00361980676802180
953	603,305,570	6,496,620	596,682.571428597	2185.71428571455	0.00366311065610890
954	603,979,670	6,499,192	585,521	2148.28571428545	0.00366901565321389
955	604,632,608	6,501,909	575,292.214285731	2101.92857142864	0.00365367115916620
956	605,009,189	6,502,953	560,739.714285672	2006.78571428591	0.00357881859115751
957	605,379,516	6,504,064	540,934.357142866	1888.71428571409	0.00349157760229909
958	605,857,593	6,505,488	524,549.142857194	1833.35714285681	0.00349511035871797
959	606,528,473	6,507,824	510,515.071428537	1793	0.00351213921066577
960	607,140,264	6,510,019	501,980.428571403	1759.21428571455	0.00350454755919696
961	607,718,057	6,512,235	500,245.142857134	1744.14285714272	0.00348657629573594
962	608,237,909	6,514,533	493,517.785714269	1727.78571428545	0.00350095936620566
963	608,551,099	6,515,431	490,361.642857134	1723.64285714319	0.00351504421736626
964	608,865,332	6,516,215	498,023.285714328	1759.57142857183	0.00353311075816069
965	609,375,209	6,517,755	495,101.428571463	1754.07142857136	0.00354285269107880
966	609,920,106	6,519,746	489,065.714285672	1727.78571428545	0.00353282935977029
967	610,613,694	6,522,228	483,507.571428537	1708.14285714272	0.00353281511620587
968	611,216,953	6,524,660	474,294.642857134	1687.21428571455	0.00355731255059267
969	611,670,433	6,526,665	469,717.857142866	1664.21428571409	0.00354300834087283
970	611,965,495	6,527,488	457,577.857142866	1620.50000000000	0.00354147381632159
971	612,220,042	6,528,072	445,788	1573.35714285728	0.00352938424286270
972	612,660,624	6,529,519	450,828.142857134	1542.64285714272	0.00342179804341004
973	613,210,741	6,531,281	453,007.428571463	1530.35714285728	0.00337821644047468
974	613,729,149	6,533,380	448,608.357142866	1523.50000000000	0.00339605799968372
975	614,342,530	6,535,535	448,094.142857134	1515.07142857136	0.00338114535242745
976	614,856,450	6,537,387	453,835.071428597	1501.64285714272	0.00330878539733786
977	615,121,582	6,538,191	458,878.071428537	1507.57142857136	0.00328534206020813
978	615,344,472	6,538,698	463,362.428571403	1534.21428571455	0.00331104593534850
979	615,809,512	6,540,104	462,221.571428597	1515.42857142864	0.00327857604469838
980	616,415,544	6,541,719	455,915.285714328	1476.71428571409	0.00323901025472390
981	616,948,639	6,544,048	452,592.285714269	1470.92857142864	0.00325000804887175
982	617,610,114	6,546,346	440,615.642857134	1471.85714285728	0.00334045594321878
983	618,059,968	6,547,792	434,065.571428597	1468.64285714272	0.00338345852288889
984	618,300,878	6,548,460	441,354.142857134	1458.71428571409	0.00330508800998447
985	618,501,468	6,549,022	446,275.428571403	1426.78571428591	0.00319709673206360
986	618,821,135	6,550,386	452,939.428571403	1430.28571428591	0.00315778584080683
987	619,480,839	6,551,998	456,564.571428597	1458.85714285681	0.00319529204443530
988	620,062,302	6,554,191	456,294.928571463	1466.57142857136	0.00321408662849444
989	620,744,307	6,556,178	470,747.857142866	1471.85714285728	0.00312663588484607
990	621,266,927	6,557,984	483,509.571428537	1494.35714285728	0.00309064645492368
991	621,485,823	6,558,692	481,479.428571403	1494	0.00310293630702530
992	621,704,652	6,559,322	476,238.142857134	1484.14285714272	0.00311638804955602
993	622,208,421	6,560,692	467,783.142857134	1484.42857142864	0.00317332634596881
994	622,862,687	6,562,613	462,862.928571463	1476.92857142864	0.00319085517603860
995	623,421,166	6,564,492	460,884.428571463	1475.64285714272	0.00320176331779436
996	624,052,777	6,566,655	461,192.357142866	1500.78571428545	0.00325414263927306
997	624,507,421	6,568,289	460,151.500000000	1529.14285714319	0.00332312913712808
998	624,725,410	6,569,064	453,735.714285672	1553.78571428591	0.00342442894699634
999	624,917,447	6,569,609	446,224	1577.21428571409	0.00353457968579477
1000	625,452,319	6,571,416	438,873.642857194	1601	0.00364797482386281
1001	626,060,910	6,573,297	433,327.714285672	1617.35714285728	0.00373241103566026
1002	626,575,243	6,575,561	428,533.500000000	1609.07142857136	0.00375483230265863
1003	627,145,836	6,577,667	418,565.642857194	1582.07142857136	0.00377974507838698
1004	627,558,593	6,579,691	408,369.142857134	1570.78571428591	0.00384648483305078
1005	627,740,826	6,580,305	401,367.571428537	1606.07142857136	0.00400149773648896
1006	627,901,500	6,580,895	388,449.714285672	1619.78571428545	0.00416987232765535
1007	628,328,185	6,582,279	375,550.500000000	1577.14285714272	0.00419954934727214
1008	628,902,212	6,584,425	372,232.714285731	1540.71428571455	0.00413911573750575
1009	629,353,087	6,586,918	373,892.785714269	1544.78571428591	0.00413162749672964
1010	629,806,288	6,588,987	365,372.428571463	1532.28571428545	0.00419376393636596
1011	630,155,848	6,590,451	342,730.714285731	1563.71428571409	0.00456251576101940
1012	630,354,829	6,591,115	325,204.714285672	1557.78571428591	0.00479016953277466
1013	630,521,996	6,591,712	322,624.571428597	1519.92857142864	0.00471113704916622
1014	630,822,903	6,592,914	329,379.714285731	1567.57142857136	0.00475916202663143
1015	631,205,724	6,595,682	334,999.714285672	1598.78571428591	0.00477249873987220
1016	631,602,441	6,597,470	336,529.357142866	1590.85714285728	0.00472724625382954
1017	632,073,678	6,599,714	339,728.857142866	1585.50000000000	0.00466695709435496
1018	632,499,774	6,601,670	345,440.642857134	1475.28571428545	0.00427073578280593
1019	632,700,899	6,602,279	351,320.428571463	1389.57142857136	0.00395528217422946
1020	632,887,337	6,602,820	352,153.928571403	1399.85714285728	0.00397512857100908
1021	633,213,766	6,604,003	355,043.928571403	1365.71428571409	0.00384660650643805
1022	633,651,030	6,605,247	362,031.571428597	1331.78571428545	0.00367864523259711
1023	634,075,621	6,607,359	364,744.142857134	1315.71428571455	0.00360722526044757
1024	634,530,653	6,609,423	367,263.428571403	1324.21428571455	0.00360562523435981
1025	635,013,414	6,611,081	374,118.500000000	1321.42857142864	0.00353211234255627
1026	635,255,701	6,611,513	382,219.714285731	1318.35714285728	0.00344921283121395
1027	635,438,953	6,612,006	387,502.285714328	1325.35714285681	0.00342025632291078
1028	635,803,838	6,613,356	399,148.928571463	1365.71428571409	0.00342156570631899
1029	636,298,617	6,614,394	409,842.071428537	1410.42857142864	0.00344139528309643
1030	636,779,110	6,616,669	412,952.642857134	1411.78571428591	0.00341875936310290
1031	637,252,196	6,618,668	415,192.285714269	1392.71428571455	0.00335438382078468
1032	637,879,956	6,620,956	419,691.571428537	1413.21428571409	0.00336726868472440
1033	638,126,948	6,621,384	432,892	1442.85714285681	0.00333306492810403
1034	638,349,043	6,621,900	436,688.857142866	1388	0.00317846443136035
1035	638,706,440	6,622,960	436,914.500000000	1322.50000000000	0.00302690801060619
1036	639,271,697	6,624,575	443,156	1302.14285714272	0.00293833967528979
1037	639,866,518	6,626,688	447,647.214285731	1304.35714285728	0.00291380600890931
1038	640,278,432	6,628,081	455,320.428571463	1311.50000000000	0.00288038910117594
1039	640,970,523	6,630,058	471,554.642857134	1310.07142857136	0.00277819643686187
1040	641,240,565	6,630,512	477,983.357142806	1263.64285714319	0.00264369635105444
1041	641,502,487	6,631,033	495,467	1288.57142857136	0.00260072099367135
1042	641,927,482	6,632,188	520,788.142857194	1365.64285714272	0.00262226180813260
1043	642,652,420	6,633,688	525,693.500000000	1386.07142857136	0.00263665316115067
1044	643,177,562	6,635,266	527,320	1396.57142857136	0.00264843250506592
1045	643,903,926	6,637,543	533,535.571428597	1419.07142857183	0.00265975036073437
1046	644,636,063	6,639,715	529,175.285714269	1501.28571428591	0.00283702915615100
1047	644,934,734	6,640,260	–	–	–
1048	645,190,798	6,640,837	–	–	–
1049	645,708,669	6,642,251	–	–	–
1050	646,279,687	6,644,643	–	–	–

To estimate the long term trends we calculated average values of *DV*, *DD*, *CFI* for every year of the pandemic with the use of accumulated characteristics on January 22 and December 31, 2020; January 1 and December 31, 2021; and January 1 and December 6, 2022. The results are listed in Table 1 and shown in Fig. 1 by dashed lines.

The *DV* values approximately doubled every year, the *DD* figure in 2021 was 1.8 times higher than in 2020. But in 2022 we see a sufficient decrease in mortality and much lower *CFR* values*.* This can be explained as a positive effect of vaccinations (the global VC values tend to some saturation in 2022, see green line), lower pathogenicity of the Omicron strain (which began to spread widely at the end of 2021^29,30^), as well as the influence of natural immunity^24^.

To simulate the re-infections, we add terms $\pm \delta R$ to the first and last differential equations of the classical SIR-model^11–17^, which relates the numbers of susceptible *S*(*t*), infectious *I*(*t*) and removed persons *R*(*t*) over time *t*:

1 $$\frac{dS}{{dt}} = - \alpha_{{}} SI + \delta R,$$

2 $$\frac{dI}{{dt}} = \alpha SI - \rho_{{}} I,$$

3 $$\frac{dR}{{dt}} = \rho_{{}} I - \delta R.$$

Now compartment *S*(*t*), which includes the persons who are sensitive to the pathogen, is increasing with rate $\delta R$ due to the persons who have lost their immunity and can be re-infected. The same term with opposite sign appears in Eq. (3).

Parameters $\alpha$ (primal infection rate), $\rho_{{}}$ (removal rate) and $\delta$ (re-infection rate) can be supposed to be constant for every epidemic wave. Corresponding generalized SIR model, initial conditions and parameter identification procedures (at $\delta$ = 0) were successfully used in^9,11,31^ to simulate and predict different waves of COVID-19 pandemic in different countries and worldwide.

The set of differential Eqs. (1)–(3) has the non-trivial equilibrium point^32–34^ $(S_{*} ,I_{*} ,R_{*} )$ corresponding to zero values of the derivatives:

4 $$S_{*} = \rho /\alpha ,\quad I_{*} = \delta R_{*} /\rho ,\quad R_{*} = \rho I_{*} /\delta .$$

At the equilibrium, the daily number of new cases DV* must be equal to the daily number of new infections; to the sum of immunized, isolated and dead persons; and to the number of persons who have lost their immunity and moved from compartment *R* to *S,* i.e.:

5 $$DV_{*} = \alpha S_{*} I_{*} = \rho I_{*} = \delta R_{*}$$

The Jacobian matrix^32–34^ of set (1)–(3) can be written as follows:

6 $$J = \left\| \begin{gathered} - \alpha I\quad - \alpha S\quad \delta \hfill \\ \alpha I\;\;\;\alpha S - \rho \quad 0 \hfill \\ 0\quad \quad \;\rho \quad \; - \delta \hfill \\ \end{gathered} \right\|$$

Taking into account (4), the eigenvalues of (6) at the equilibrium point can be calculated as follows:

$$\lambda_{1} = 0,\lambda_{2,3} = \frac{{ - (\alpha I_{*} + \delta ) \pm \sqrt {(\alpha I_{*} + \delta )^{2} - 4\alpha I_{*} (\rho + \delta )} }}{2}$$

Eigenvalues $\lambda_{2,3}$ have negative real parts, but the presence of a zero eigenvalue $\lambda_{1}$ indicates that the system can approach no equilibrium with increasing time. Its asymptotic stability needs further investigations with the use of non-linear methods.

To estimate the endemic global number of new daily cases we can use the average *DV* value for 2022 (around 1 million), presented in Table 1. More optimistic prediction $DV \approx 300,000$ follows from the minimum of *DV*_*i*_ values illustrated by the blue solid line and non-linear correlation approach^35^, which yields the best fitting curve

7 $$DC \approx \frac{{1.2484 \cdot 10^{8} }}{{(t - 674.5)^{0.9684} }},\quad t > 710$$

for the 2022 dataset (shown by the dotted blue line). Thus, the global daily numbers of SARS-CoV-2 cases can vary from 300 thousand to one million and the daily numbers of deaths from 1.0 to 3.3 thousand (if we use the *CFR* value from the Table 1 corresponding to 2022). According to the recent WHO report “nearly 2.8 million new cases and over 13,000 deaths were reported in the week of 9–15 January 2023. In the last 28 days (19 December 2022–15 January 2023), nearly 13 million cases and almost 53,000 new deaths were reported globally”^7^. Calculating the average daily characteristics, we obtain approximately 400,000–464,000 new daily cases and 1857–1893 daily deaths. These figures ​​are consistent with the given theoretical estimates of SARS-CoV-2 endemic characteristics.

## Discussion

It is necessary to emphasize that the presented estimates refer to the laboratory confirmed cases only. Due to the high numbers of asymptomatic patients and insufficient testing level, the real number of SARS-CoV-2 cases is much higher^35–37^. To estimate the global visibility coefficient let us take the number of cases per million 72,524 registered worldwide as of August 1, 2022^8^. On the same day, the average value was 460,834 for European countries with high enough testing level (more than 3 tests per capita)^35^. It means that the real numbers of cases can be approximately 6 times higher than registered figures. In the future, reducing the testing level can lead to lower numbers of detected cases and deaths.

Other restrictions on the accuracy of the given estimates may be related to the situation in mainland China, where the Zero-COVID policy^38^ began to be lifted only after November 30, 2022^39^. Very high numbers of new daily cases in December 2022 reported by the media^40^ do not look reliable^41^, nevertheless it is better to use the presented estimations excluding the Chinese datasets.

According to the presented estimations, the annual mortality caused by SARS-CoV-2 will range from 365 thousand to 1.2 million. Unfortunately, these figures are higher than in the case of seasonal influenza (between 294 and 518 thousand annual deaths in the period from 2002 to 2011^42^). 

## Conclusions

Thus, the proposed modified SIR model with re-infections allowed us to make adequate estimations of the equilibrium (endemic) global daily numbers of SARS-CoV-2 cases and related deaths. In different countries and regions, these characteristics are different and need special investigations. To take into account the influence of other factors (e.g., vaccination and/or testing rates), more complicated mathematical models can be used.

## Methods

Since numbers of new cases and deaths are random and characterized by some weekly periodicity, the use of the smoothed characteristics is recommended^11^:

8 $$\overline{C}_{i} = \frac{1}{7}\sum\limits_{j = i - 3}^{j = i + 3} {C_{j} } ,\quad C_{j} = V_{j} ;D_{j}$$

Some different smoothing procedure is used in^8^ based on the values corresponding to the fixed and previous 6 days. To estimate the smoothed numbers of new daily cases *DV*_*i*_, deaths *DD*_*i*_, and the case fatality risk *CFR*_*i*_ =*DD*_*i*_*/DV*_*i*_, the numerical derivatives of the smoothed values (8) are calculated as follows^11^:

9 $$\left. {DC_{i} \equiv \frac{{d\overline{C} }}{dt}} \right|_{{t = t_{i} }} \approx \frac{1}{2}\left( {\overline{C}_{i + 1} - \overline{C}_{i - 1} } \right),\quad C_{j} = V_{j} ;\,D_{j}$$

The results of calculations are listed in Table 2.

### Human/animals involved

No humans or human data was used during this study.

## Data Availability

All data generated or analysed during this study are included in this published article.
